# Ilexoside K Ameliorates Atherosclerosis by Suppressing CD72^hi^ Macrophages and Blocking CXCL12–CXCR4 Axis to Inhibit Endothelial Pyroptosis

**DOI:** 10.1002/advs.77982

**Published:** 2026-09-27

**Authors:** Xingling He, Sijing Li, Jiahui Chen, Liyu Lin, Xinyu Li, Xiaojiao Zhang, Yihui Zhang, Xingling Chen, Jinjian Guo, Zhongqi Yang, Lu Lu, Shihao Ni

**Affiliations:** ^1^ State Key Laboratory of Traditional Chinese Medicine Syndrome The First Affiliated Hospital of Guangzhou University of Chinese Medicine Guangzhou China; ^2^ Fujian University of Traditional Chinese Medicine Fuzhou China; ^3^ The Second Affiliated Hospital of Fujian University of Traditional Chinese Medicine Fuzhou China; ^4^ Guangzhou University of Chinese Medicine Guangzhou China; ^5^ Guangdong Clinical Research Institute of Traditional Chinese Medicine Department of Geriatrics The First Affiliated Hospital of Guangzhou University of Chinese Medicine Guangzhou China; ^6^ Department of Neurology The First Affiliated Hospital of Jinan University Guangzhou China

**Keywords:** atherosclerosis, CD72^hi^ macrophages, CXCL12‐CXCR4 axis, endothelial pyroptosis, Ilexoside K, vascular inflammation

## Abstract

Atherosclerotic cardiovascular disease (ASCVD) remains the leading global cause of mortality, with substantial residual inflammatory risk persisting despite statin therapy. We identify a c‐Rel‐driven CD72^hi^ macrophage subset markedly enriched in human and murine atherosclerotic plaques, whose abundance correlates with disease severity. Single‐cell transcriptomics and functional assays demonstrate that CD72^hi^ macrophages drive endothelial pyroptosis, oxidative stress and dysfunction via pathological crosstalk through the CXCL12–CXCR4 axis. Through transcriptome‐based phenotypic screening, we discover Ilexoside K (IK), a natural triterpenoid saponin, as a dual anti‐atherosclerotic and anti‐pyroptotic lead compound. Molecular docking, dynamics simulations, and surface plasmon resonance (SPR) validate that IK directly binds CXCR4. In vivo, IK significantly alleviates atherosclerotic plaque burden, dyslipidemia, systemic inflammation and endothelial pyroptosis in high‐fat diet‐fed ApoE^−/−^ mice with favorable safety. Gain‐of‐function assays confirm that endothelial CXCR4 overexpression abrogates IK's vasculoprotective effects. This study defines CD72^hi^ macrophages as a key pro‐atherogenic subset and establishes IK as a promising CXCR4‐associated agent for ASCVD treatment.

## Graphical abstract

CD72^hi^ macrophages drive endothelial pyroptosis and accelerate atherosclerosis through CXCL12–CXCR4‐mediated immune–vascular crosstalk. Ilexoside K suppresses this pathogenic communication by modulating CXCR4‐dependent endothelial responses, reducing inflammation and plaque progression. These findings reveal a macrophage–endothelial regulatory axis and identify a potential therapeutic strategy for targeting residual inflammatory risk in atherosclerosis.

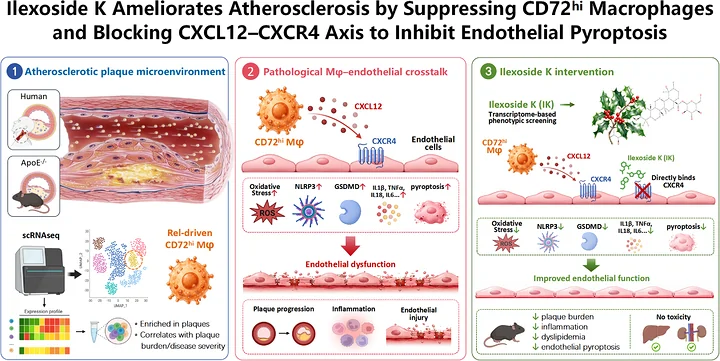

## 1 Introduction

Atherosclerotic cardiovascular disease (ASCVD) remains the leading cause of global mortality and disability [1, 2, 3], pathologically characterized by chronic vascular inflammation [4, 5], endothelial dysfunction, and lipid deposition within the arterial intima [6], culminating in plaque formation, acute cardiovascular events, and ultimately fatal outcomes. Despite the broad clinical implementation of statin‐centric lipid‐lowering therapies, antiplatelet regimens, and other standard‐of‐care interventions that have markedly reduced the risk of acute cardiovascular events, over 50% of treated individuals still face significant residual cardiovascular risk [7, 8]. The core driver of this residual risk is rooted in persistent chronic vascular inflammation [4, 5], endothelial injury [6], and disrupted immune homeostasis within the atherosclerotic microenvironment [9]—pathological processes that are not adequately targeted by current lipid‐focused treatment paradigms. Thus, delineating the core immuno‐vascular crosstalk mechanisms governing atherosclerotic initiation and progression, alongside identifying novel therapeutic targets and translational lead compounds, remain critical unmet needs in contemporary cardiovascular research.

Macrophages are the most prevalent innate immune cells within atherosclerotic plaques [10, 11, 12], and their functional heterogeneity acts as a central regulatory hub governing plaque initiation, progression, instability, and ultimate rupture [13, 14, 15, 16]. During atherogenesis, macrophages in the vascular niche become hyperactivated, releasing excessive pro‐inflammatory cytokines such as interleukins (ILs), tumor necrosis factor (TNF), and chemokines [17], which subsequently trigger endothelial cell activation, lipid accumulation, and vascular smooth muscle cell phenotypic switching [18, 19, 20]. These inflammatory mediators further recruit circulating monocytes to the lesion site, differentiate into pro‐inflammatory macrophage subsets, and exacerbate endothelial injury and local inflammatory cascades, thereby establishing a vicious pathological cycle that drives plaque progression [21, 22]. Recent advances in single‐cell transcriptomics have resolved a remarkably complex landscape of macrophage subpopulations in the atherosclerotic vascular niche [23, 24]. However, the phenotypic signatures, upstream transcriptional regulatory networks, and downstream pathological effector mechanisms of macrophage subpopulations with definitive pro‐atherogenic activity in atherogenesis remain incompletely defined. In our prior work [25], we identified CD72^hi^ macrophages as a c‐Rel‐driven pro‐inflammatory macrophage population endowed with robust tissue‐injurious potential, yet the functional contribution of CD72^hi^ macrophages to atherogenesis, and the molecular mechanisms underpinning their putative pro‐atherogenic activity, remain entirely uncharacterized.

Cell‐cell crosstalk between macrophages and vascular endothelial cells constitutes a pivotal mechanism underlying atherosclerotic progression [26, 27]. Emerging evidence has established that pyroptosis, a pro‐inflammatory form of regulated cell death mediated by the NLRP3–caspase‐1–GSDMD axis, is a critical driver of endothelial damage and barrier dysfunction in atherosclerosis [28, 29, 30]. However, most existing therapeutic strategies targeting pyroptosis focus on directly inhibiting the core pyroptotic machinery, which may lead to off‐target effects due to the broad physiological roles of these molecules in innate immunity, and lack cell‐type specificity for the vascular endothelium [31, 32]. In this context, modulating the upstream intercellular drivers of endothelial pyroptosis in the plaque microenvironment represents a more specific and safer therapeutic strategy. Through cell‐cell communication analysis based on single‐cell transcriptomics, we identified that the CXCL12–CXCR4 axis exhibits the strongest predicted interaction between CD72^hi^ macrophages and endothelial cells in atherosclerotic lesions, suggesting that this signaling axis may serve as the core mediator of the pathogenic macrophage‐endothelial crosstalk that drives endothelial pyroptosis and atherogenesis.

Natural products have long represented an invaluable source for the discovery of bioactive molecules with anti‐inflammatory and vasculoprotective activities, offering a robust pipeline for translational cardiovascular drug discovery and development [33, 34]. Saponins from *Ilex* species have been reported to exhibit hypocholesterolemic and anti‐inflammatory activities; however, the specific bioactive constituent responsible for these anti‐atherosclerotic effects and its underlying mechanism remain unclear [35]. Leveraging a transcriptome‐based phenotypic screening platform we previously established [36, 37, 38], we performed a screen of 102 phytochemical compounds and identified Ilexoside K (IK), a natural triterpenoid saponin, as a high‐potential lead compound with dual anti‐atherosclerotic and anti‐pyroptotic activities. Notably, we demonstrate that IK exerts a potent inhibitory effect on CD72^hi^ macrophage‐induced endothelial pyroptotic injury; yet, its anti‐atherosclerotic efficacy and the underlying molecular mechanisms have not been reported in any prior study.

In this study, we integrated single‐cell transcriptomics, genetic depletion and gain‐of‐function models, transcriptome‐based drug screening, and molecular target validation to systematically elucidate the pathogenic role of CD72^hi^ macrophages in atherogenesis. We first demonstrated that c‐Rel‐driven CD72^hi^ macrophages are markedly enriched in atherosclerotic plaques from humans and ApoE^−/−^ mice, whose abundance positively correlates with plaque burden and disease severity. Functional assays revealed that CD72^hi^ macrophages drive endothelial pyroptosis, oxidative stress and dysfunction via pathological crosstalk with endothelial cells through the CXCL12–CXCR4 axis. Furthermore, we identified IK as a CXCR4‐modulating lead compound, which was validated via molecular docking, molecular dynamics simulations and surface plasmon resonance (SPR) analysis. In vivo, IK significantly alleviated atherosclerotic plaque burden, dyslipidemia, systemic inflammation and endothelial pyroptosis in high‐fat diet‐fed ApoE^−/−^ mice, with a favorable safety profile. Gain‐of‐function assays confirmed that endothelial CXCR4 overexpression abrogated the vasculoprotective effects of IK. Collectively, our study defines CD72^hi^ macrophages as a key pro‐atherogenic subset, and identifies IK as a CXCR4‐modulating lead compound for ASCVD treatment, providing an innovative therapeutic strategy targeting immuno‐vascular crosstalk for the management of ASCVD.

## 2 Results

### 2.1 CD72^hi^ Macrophages Are Expanded in Murine and Human Atherosclerosis and Associate With Disease Burden

**FIGURE 1:**
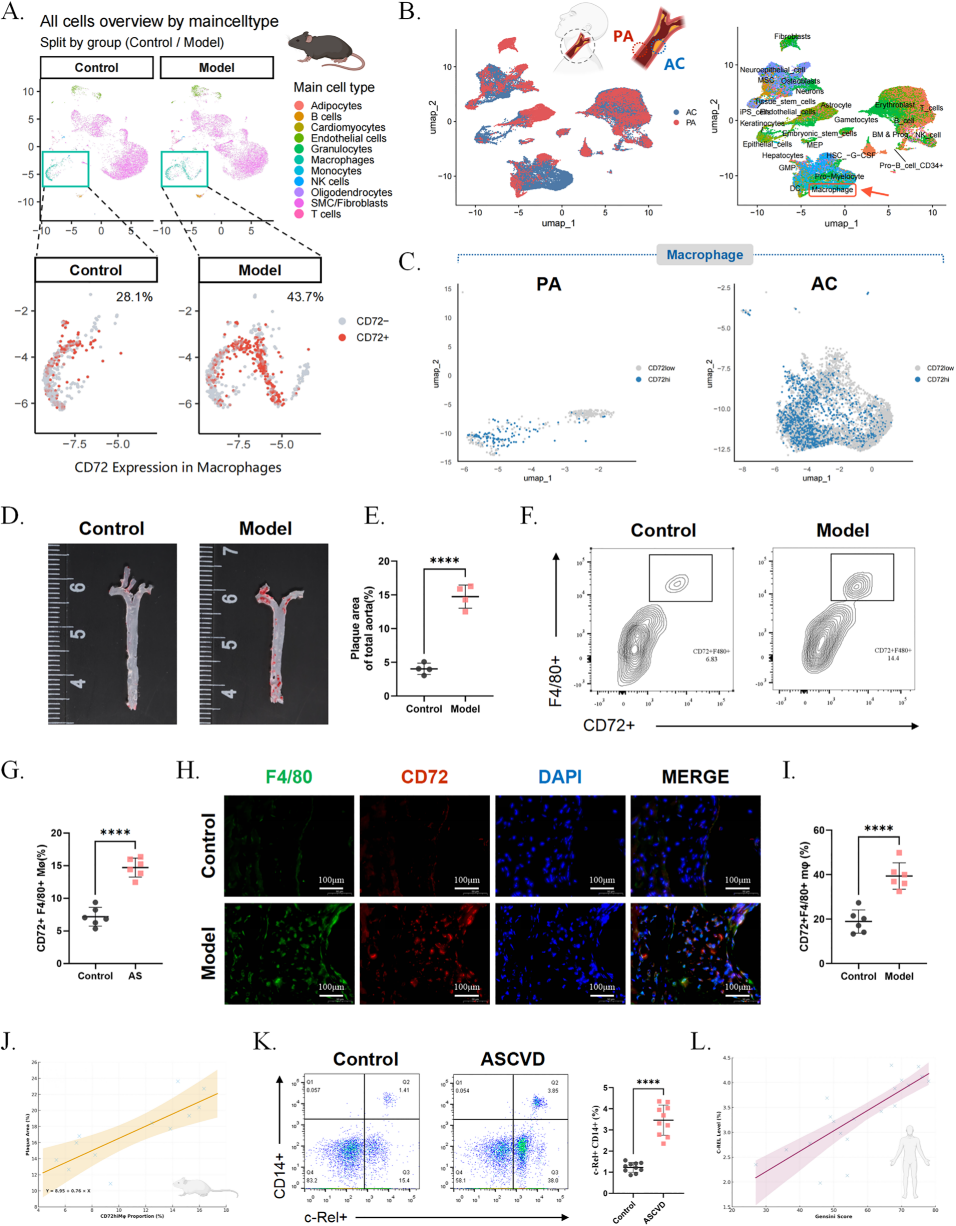
CD72^hi^ macrophages are expanded in murine and human atherosclerosis and associate with disease burden. (A) t‐distributed stochastic neighbor embedding (t‐SNE) plots of single‐cell RNA‐seq data from aortas of control and atherosclerotic ApoE^−/−^ mice. Upper panels show clustering of major cell populations with macrophage clusters outlined by blue boxes; lower panels show CD72 expression mapped onto the macrophage clusters, with the proportions of CD72‐positive macrophages indicated for each group. (B) UMAP plots of a published single‐cell dataset from human carotid specimens including plaque‐adjacent arterial tissue (PA) and atherosclerotic plaque (AC). The left panel displays combined PA and AC; the right panel shows cell type–annotated Uniform Manifold Approximation and Projection (UMAP) plots with macrophage clusters indicated by arrows.(C) UMAP plots of reclustered macrophages from PA and AC samples, showing CD72^hi^ macrophages in PA (left) and AC (right), with the proportions of CD72^hi^ cells indicated. (D) Representative en‐face Oil Red O staining of aortas from control and atherosclerotic ApoE^−/−^ mice. (E) Quantification of Oil Red O‐positive aortic plaque area (*n = *4 per group). (F) Representative flow cytometry plots of CD72 and F4/80 expression in aortic cell suspensions from control and model mice. (G) Quantification of CD72^+^F4/80^+^ macrophages in aortic cell suspensions from control and model mice (*n* = 6 per group). (H) Immunofluorescence staining of aortic root sections for CD72 (red) and F4/80 (green), with nuclei counterstained by 4′,6‐diamidino‐2‐phenylindole (DAPI) (blue). Scale bars, 100 µm. (I) Quantification of CD72^+^F4/80^+^ macrophages in aortic root lesions (*n* = 6 per group). (J) Correlation between aortic plaque area and the proportion of CD72^hi^ macrophages in aortic tissue; correlation coefficients and *p* values are shown in the panel. (K) Representative flow cytometry plots and quantification of circulating c‐Rel^+^CD14^+^ monocytes in control subjects and patients with atherosclerotic coronary disease (*n* = 10 per group). (L) Correlation between the frequency of circulating c‐Rel^+^CD14^+^ monocytes and the Gensini score in patients with coronary atherosclerosis; correlation coefficients and *p* values are shown in the panel. Data presentation and statistical tests are described in the Methods. Data are presented as mean ± standard deviation (SD) (or mean ± SEM where indicated); statistical analyses were performed as described in the Methods. ^*^ *p* < 0.05; ^**^ *p* < 0.01; ^***^ *p* < 0.001.

We first asked whether CD72^hi^ macrophages are enriched during atherosclerosis. Single‐cell RNA sequencing (scRNA‐seq) of aortas from control and atherosclerotic ApoE^−/−^ mice resolved the major immune and stromal cell populations, with macrophages forming a distinct cluster on the t‐SNE map (Figure 1A, upper panels). When CD72 expression was projected onto the macrophage compartment, the fraction of CD72^hi^ cells increased from 28.1% in control aortas to 43.7% in atherosclerotic aortas (Figure 1A, lower panels), indicating expansion of this macrophage subset during atherosclerotic progression.

To determine whether this phenotype extends to human disease, we re‐analysed a published single‐cell dataset from human carotid specimens comprising plaque‐adjacent arterial tissue (PA) and atherosclerotic plaque (AC). UMAP embedding separated PA and AC samples and delineated the major vascular and immune cell types, with macrophages forming a discrete cluster (Figure 1B). Reclustering of this macrophage cluster revealed that CD72^hi^ cells accounted for 28.03% of macrophages in PA but 33.88% in AC lesions (Figure 1C), suggesting an enrichment of the CD72^hi^ macrophage phenotype in human atherosclerotic lesions.

We next validated the expansion of CD72^hi^ macrophages at the protein, cellular, and tissue levels in the murine atherosclerosis model. Consistent with the single‐cell findings, Western blot analysis of aortic tissues demonstrated that CD72 protein expression was markedly increased in ApoE^−/−^ mice compared with control mice (Figure S1A,B), supporting the upregulation of CD72 during atherosclerotic development. En‐face Oil Red O staining showed extensive lipid deposition in aortas from ApoE^−/−^ model mice compared with controls (Figure 1D,E). Flow cytometric analysis of aortic cell suspensions demonstrated a significantly increased proportion of CD72^+^F4/80^+^ macrophages in ApoE^−/−^ mice compared with controls (Figure 1F,G). This finding was further supported by aortic root immunofluorescence staining, which showed increased CD72 and F4/80 signals and enhanced co‐localization within atherosclerotic plaques (Figure 1H,I). Importantly, the proportion of aortic CD72^hi^ macrophages positively correlated with plaque area (Figure 1J), linking expansion of this subset to lesion burden in vivo.

Given our previous findings that c‐Rel signaling promotes the CD72^hi^ macrophage phenotype, we further examined whether this macrophage‐associated signature was relevant in human coronary atherosclerosis. Flow cytometry of peripheral blood showed that the frequency of c‐Rel^+^CD14^+^ monocytes was significantly higher in patients with angiographically documented atherosclerotic coronary disease than in control subjects (Figure 1K). Moreover, circulating c‐Rel^+^CD14^+^ monocytes strongly correlated with coronary atherosclerotic burden, as reflected by the Gensini score (Figure 1L). Together, these data demonstrate that CD72^hi^ macrophages are expanded in both murine and human atherosclerosis and closely track with structural plaque burden and clinical disease severity.

### 2.2 Rel‐Dependent CD72^hi^ Macrophages Expansion Contributes to Endothelial Pyroptosis and Atherosclerotic Progression

**FIGURE 2:**
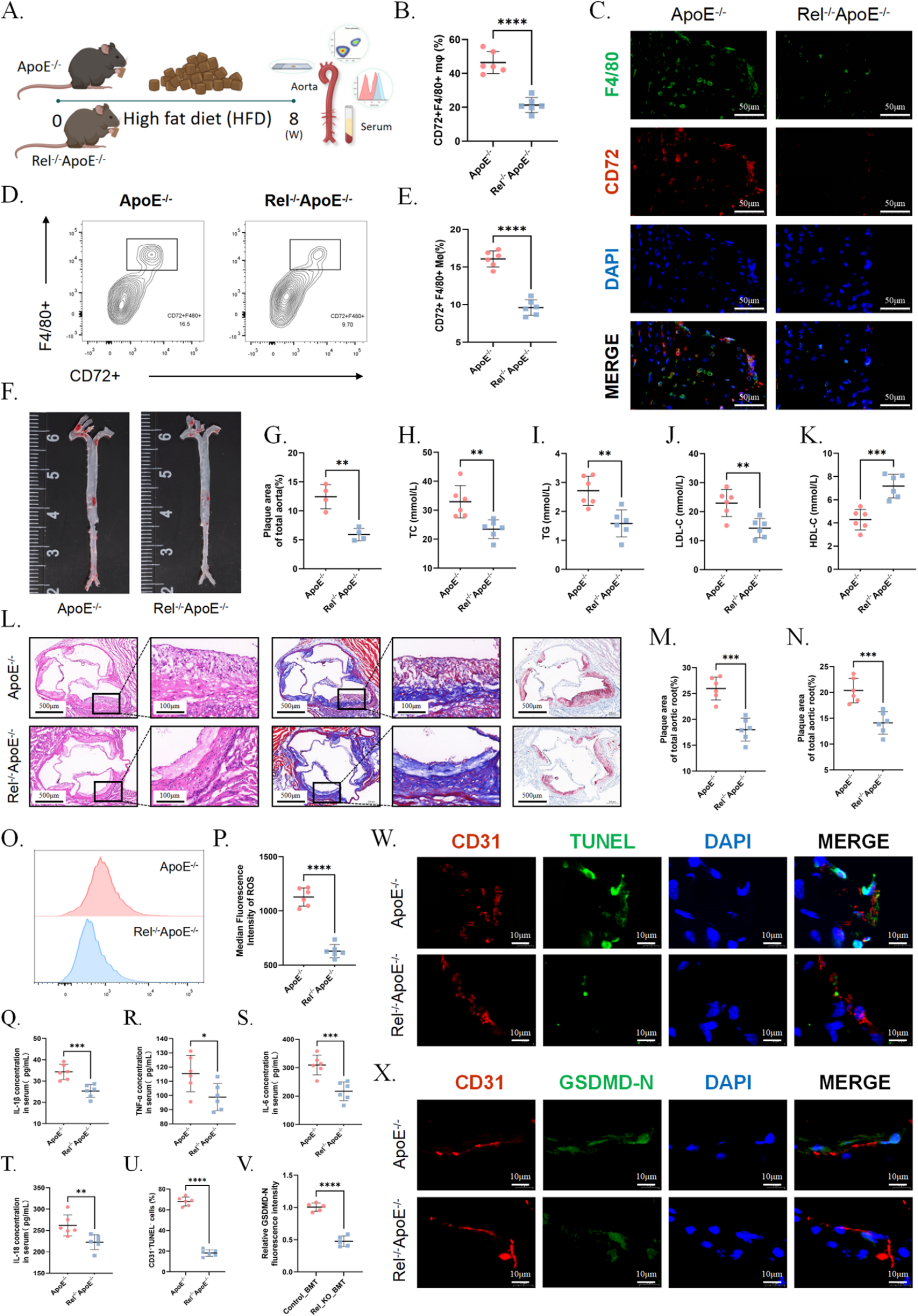
Global Rel deficiency reduces CD72^hi^ macrophage accumulation and attenuates atherosclerosis‐associated inflammation and endothelial injury in ApoE^−/−^ mice. (A) Schematic diagram of the experimental protocol for ApoE^−/−^ and Rel^−/−^ApoE^−/−^ mice. (B, C) Quantification of CD72^+^F4/80^+^ macrophages in aortic root lesions and representative immunofluorescence images of aortic root sections stained for F4/80 (green), CD72 (red), and DAPI (blue) from ApoE^−/−^ and Rel^−/−^ApoE^−/−^ mice (*n* = 6 per group). Scale bars, 50 µm. (D, E) Representative flow cytometry plots of CD72 and F4/80 expression in aortic cell suspensions and quantification of CD72^+^F4/80^+^ macrophages in the two groups (*n* = 6 per group). (F, G) Representative en‐face Oil Red O staining of whole aortas and quantification of Oil Red O–positive area in whole aortas (*n* = 4 per group). (H–K) Serum levels of total cholesterol (H), triglycerides (I), low‐density lipoprotein cholesterol (LDL‐C) (J) and high‐density lipoprotein cholesterol (HDL‐C) (K) in the two groups (*n* = 6 per group). (L) Representative haematoxylin and eosin (H&E), Masson's trichrome, and Oil Red O staining of aortic root sections from the two groups. Black boxes indicate the regions shown at higher magnification. Scale bars are indicated in the images. (M) Quantification of plaque area in H&E‐stained aortic root sections (*n* = 6 per group). (N) Quantification of Oil Red O–positive plaque area in aortic root sections (*n* = 6 per group). (O) Representative flow cytometry histograms of ROS levels in aortic cell suspensions from the two groups. (P) Quantification of reactive oxygen species (ROS) signal in aortic cell suspensions (*n* = 6 per group). (Q–T) Serum concentrations of IL‐1β (Q), TNF‐α (R), IL‐6 (S) and IL‐18 (T) in the two groups (*n* = 6 per group). (U) Quantification of terminal deoxynucleotidyl transferase dUTP nick end labeling (TUNEL)‐positive endothelial cells in aortic root sections, expressed as the percentage of CD31^+^TUNEL^+^ cells among CD31^+^ endothelial cells (*n* = 6 per group). (V) Quantification of endothelial GSDMD‐N fluorescence intensity in aortic root sections (*n* = 6 per group). (W) Representative immunofluorescence images of aortic root sections stained for CD31 (red), TUNEL (green), and DAPI (blue) in the two groups. Scale bars, 10 µm. (X) Representative immunofluorescence images of aortic root sections stained for CD31 (red), GSDMD‐N (green), and DAPI (blue) in the two groups. Scale bars, 10 µm.

To define the functional contribution of Rel‐dependent CD72^hi^ macrophages to atherogenesis, ApoE^−/−^ mice and Rel^−/−^ApoE^−/−^ double‐knockout mice were subjected to the same 8‐week high‐fat diet protocol (Figure 2A). Immunofluorescence staining of aortic root lesions showed that both F4/80 and CD72 signals were readily detectable within plaques, whereas their co‐localization was markedly reduced in Rel^−/−^ApoE^−/−^ mice compared with ApoE^−/−^ mice. Quantitative analysis further confirmed a significant reduction in CD72/F4/80 double‐positive signals within atherosclerotic lesions (Figure 2B,C). Consistently, flow cytometric analysis of digested aortas identified a reduced proportion of CD72^+^F4/80^+^ macrophages in Rel^−/−^ApoE^−/−^ mice (Figure 2D,E). In line with these cellular findings, Western blot analysis further demonstrated that aortic CD72 protein expression was significantly decreased in Rel^−/−^ApoE^−/−^ mice relative to ApoE^−/−^ mice (Figure S1E,F), supporting the concept that Rel deficiency suppresses the expansion of CD72^hi^ macrophages in vivo.

We next examined the effect of Rel deficiency on atherosclerotic lesion formation and systemic lipid metabolism. En‐face Oil Red O staining revealed a marked reduction in aortic lipid deposition in Rel^−/−^ApoE^−/−^ mice compared with ApoE^−/−^ controls (Figure 2F,G). Serum lipid profiling showed significantly lower levels of total cholesterol (TC), triglycerides (TG), and Low‐Density Lipoprotein Cholesterol (LDL‐C), together with increased HDL‐C, in Rel^−/−^ApoE^−/−^ mice (Figure 2H–K). Histological analysis of aortic root sections by haematoxylin–eosin, Masson's trichrome, and Oil Red O staining further demonstrated reduced plaque burden and improved plaque characteristics in Rel‐deficient mice (Figure 2L). Plaque area on H&E‐stained sections and Oil Red O–positive plaque area in aortic roots were quantified separately (Figure 2M,N).

To assess oxidative stress and inflammatory activation, we measured reactive oxygen species (ROS) levels in aortic cell suspensions and circulating cytokines. Representative ROS flow cytometry histograms and quantitative analysis showed that Rel deficiency markedly reduced ROS accumulation in aortic cells (Figure 2O,P). In parallel, serum concentrations of IL‐1β, TNF‐α, IL‐6, and IL‐18 were all significantly decreased in Rel^−/−^ApoE^−/−^ mice compared with ApoE^−/−^ mice (Figure 2Q–T), indicating reduced circulating inflammatory activation.

Finally, we examined endothelial injury and pyroptotic activation in aortic root lesions. CD31/TUNEL double immunofluorescence staining and quantitative analysis demonstrated a significant reduction in CD31^+^TUNEL^+^ endothelial cells in Rel^−/−^ApoE^−/−^ mice (Figure 2U,W). More importantly, CD31/GSDMD‐N double immunofluorescence staining revealed marked attenuation of endothelial pyroptotic signaling in the Rel‐deficient group, accompanied by reduced GSDMD‐N fluorescence intensity (Figure 2V,X). In addition, CD31/CASP1 double immunofluorescence staining provided complementary evidence that endothelial caspase‐1 activation was reduced in Rel^−/−^ApoE^−/−^ mice (Figure S1C,D). Collectively, these results indicate that genetic disruption of Rel signaling suppresses CD72^hi^ macrophage expansion, alleviates atherosclerotic progression, and attenuates endothelial inflammatory injury and pyroptotic activation.

To determine whether the protective effects of Rel deficiency were mediated by hematopoietic cells, we performed bone marrow transplantation experiments in ApoE^−/−^ recipients reconstituted with control or Rel‐deficient bone marrow (Figure S2A). Consistent with the global Rel‐deficient model, transplantation of Rel‐deficient bone marrow reduced aortic CD72 expression and CD72^+^F4/80^+^ macrophage accumulation, as confirmed by Western blot, flow cytometry, and immunofluorescence analyses (Figure S2B–G). Moreover, Rel_KO_BMT mice displayed reduced atherosclerotic lesion formation, inflammatory responses, and endothelial pyroptotic responses compared with Control_BMT mice (Figure S2H–X). These findings support the contribution of hematopoietic Rel signaling to CD72^hi^ macrophage expansion and macrophage‐associated vascular injury during atherosclerosis.

Together, these findings demonstrate that Rel‐dependent signaling promotes CD72^hi^ macrophage expansion and contributes to endothelial inflammatory injury, pyroptotic activation, and atherosclerotic progression. The bone marrow transplantation experiments further indicate that hematopoietic Rel signaling represents an important component of this regulatory axis.

### 2.3 CD72^hi^ Macrophages Enhance Macrophage–Endothelial Crosstalk and Promote Endothelial Inflammatory and Pyroptotic Activation

**FIGURE 3:**
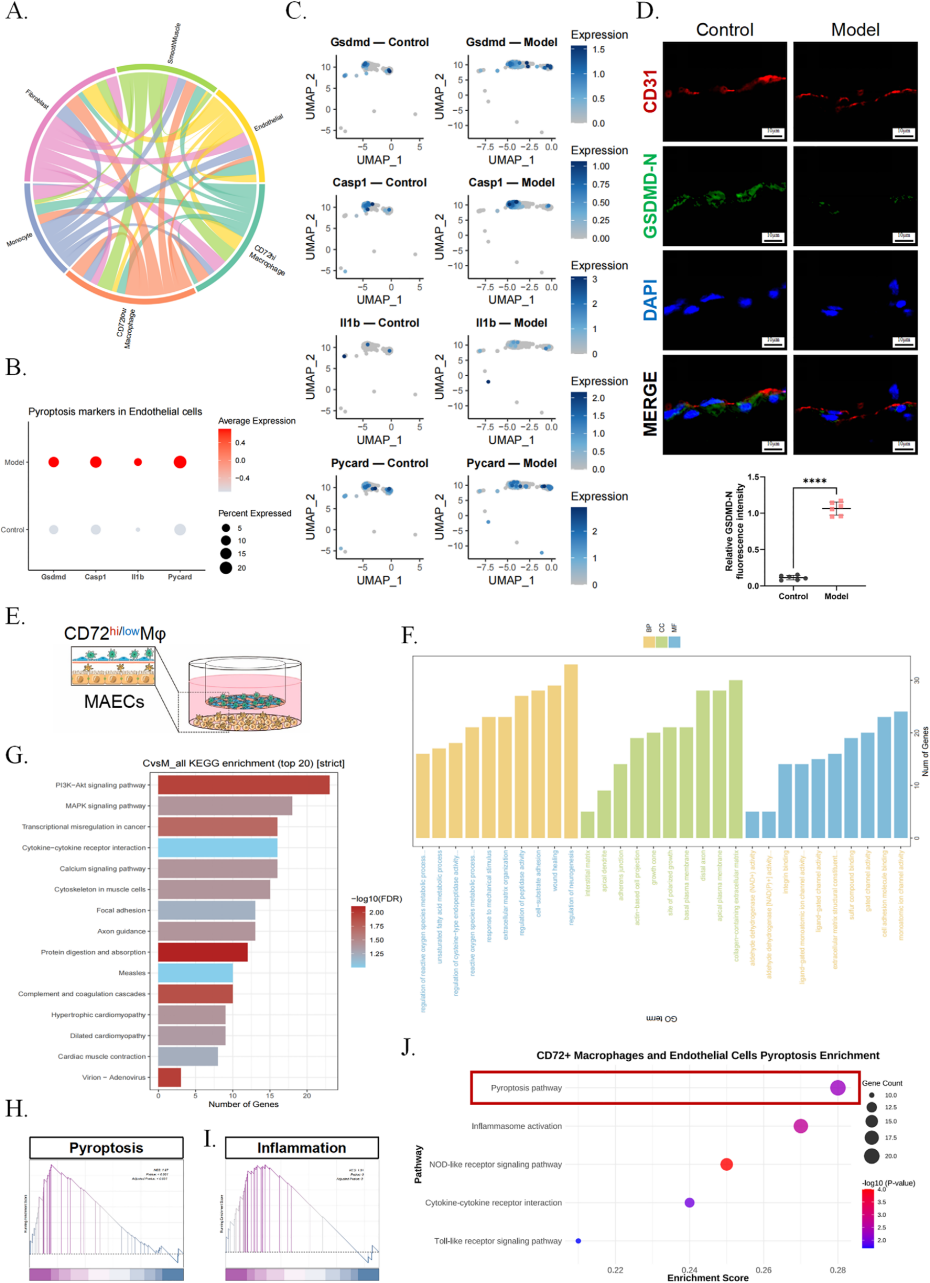
CD72^hi^ macrophages enhance macrophage–endothelial crosstalk and promote endothelial pyroptosis. (A) Circos plot of predicted cell–cell communication between CD72^hi^ or CD72^low^ macrophages and endothelial cells based on murine aortic single‐cell RNA‐seq data. (B) Bubble plot showing endothelial expression of *Gsdmd*, *Casp1*, *Il1b*, and *Pycard* in control and atherosclerotic (model) mice. (C) UMAP feature plots of endothelial clusters displaying expression of *Gsdmd*, *Casp1*, *Il1b*, and *Pycard* in control and model aortas. (D) Representative immunofluorescence images of aortic root sections stained for CD31 (red), GSDMD‐N (green), and DAPI (blue), together with quantification of endothelial GSDMD‐N fluorescence intensity in control and model mice (*n* = 6 per group). Scale bars, 10 µm. (E) Schematic illustration of the transwell co‐culture system with sorted CD72^hi^ or CD72^low^ macrophages in the upper chamber and mouse aortic endothelial cells (MAECs) in the lower chamber. (F) Gene Ontology enrichment plots (Biological Process, Cellular Component, Molecular Function) of differentially expressed genes in MAECs after co‐culture with CD72^hi^ versus CD72^low^ macrophages. (G) Kyoto Encyclopedia of Genes and Genomes (KEGG) pathway enrichment plot (top pathways) of differentially expressed genes in MAECs after co‐culture with CD72^hi^ versus CD72^low^ macrophages. (H) gene set enrichment analysis (GSEA) plot for a pyroptosis‐related gene set in MAECs co‐cultured with CD72^hi^ versus CD72^low^ macrophages. (I) GSEA plot for an inflammation‐related gene set in MAECs co‐cultured with CD72^hi^ versus CD72^low^ macrophages. (J) Bubble plot summarizing enrichment of pyroptosis‐related and innate immune signaling pathways in MAEC transcriptomes after co‐culture with CD72^hi^ macrophages.

Having established that CD72^hi^ macrophages are expanded during atherosclerosis, we next investigated whether this macrophage subset exhibits enhanced communication with endothelial cells and contributes to endothelial dysfunction. Cell–cell communication analysis based on murine single‐cell transcriptomic data revealed that CD72^hi^ macrophages exhibited stronger predicted interactions with endothelial cells compared with CD72^low^ macrophages (Figure 3A), suggesting enhanced CD72^hi^ macrophages–endothelial communication within the atherosclerotic microenvironment. We next examined whether endothelial cells exhibited a pyroptosis‐associated transcriptional phenotype during atherosclerosis. Bubble plot analysis demonstrated that canonical pyroptosis‐related genes, including Gsdmd, Casp1, Il1b, and Pycard, were markedly upregulated in endothelial cells from atherosclerotic ApoE^−/−^ aortas compared with control mice, as reflected by increased expression levels and gene detection proportions (Figure 3B). Consistently, UMAP feature plots showed enhanced expression of these pyroptosis‐related genes within endothelial clusters from atherosclerotic vessels (Figure 3C). To further validate endothelial pyroptotic activation at the tissue level, we performed immunofluorescence staining of aortic root sections. Compared with control mice, ApoE^−/−^ mice exhibited increased CD31^+^GSDMD‐N^+^ endothelial cells within atherosclerotic lesions, accompanied by enhanced GSDMD‐N fluorescence intensity (Figure 3D). Quantitative analysis further confirmed increased GSDMD‐N fluorescence intensity in atherosclerotic plaques. In parallel, CD31/CASP1 double immunofluorescence staining showed enhanced endothelial caspase‐1 activation in ApoE^−/−^ mice, providing complementary evidence of endothelial inflammasome activation (Figure S3A,B).

To investigate whether CD72^hi^ macrophages could induce endothelial dysfunction, we established a transwell co‐culture system in which sorted CD72^hi^ or CD72^low^ macrophages were cultured in the upper chamber and mouse aortic endothelial cells (MAECs) were cultured in the lower chamber (Figure 3E). RNA sequencing analysis of MAECs after co‐culture revealed extensive transcriptional alterations in endothelial cells exposed to CD72^hi^ macrophages compared with those co‐cultured with CD72^low^ macrophages. Gene Ontology enrichment analysis demonstrated that differentially expressed genes in MAECs exposed to CD72^hi^ macrophages were mainly associated with ROS metabolism, extracellular matrix organization, cell adhesion, junctional regulation, and cellular stress responses (Figure 3F), suggesting broad endothelial functional remodeling.

KEGG pathway analysis further revealed enrichment of signaling pathways involved in endothelial stress and inflammatory regulation, including PI3K/AKT signaling, MAPK signaling, cytokine–cytokine receptor interaction, calcium signaling, focal adhesion, and complement/coagulation cascades (Figure 3G). These results indicate that exposure to CD72^hi^ macrophages induces endothelial transcriptional reprogramming associated with inflammatory activation and vascular dysfunction. To further determine whether CD72^hi^ macrophages directly activate endothelial pyroptosis‐related programs, Gene set enrichment analysis (GSEA) was performed. A curated pyroptosis gene set showed significant enrichment in MAECs co‐cultured with CD72^hi^ macrophages compared with CD72^low^ macrophages (Figure 3H), indicating activation of pyroptosis‐associated transcriptional signatures. Similarly, inflammatory response‐related gene sets were strongly enriched in CD72^hi^ macrophage‐exposed MAECs (Figure 3I), consistent with a pro‐inflammatory endothelial phenotype.

To integrate these pathway‐level alterations, we further summarized key inflammatory and innate immune pathways using pathway enrichment analysis. Among the enriched pathways, pyroptosis‐related signaling exhibited a prominent enrichment pattern, together with inflammasome activation, NOD‐like receptor signaling, cytokine–cytokine receptor interaction, and Toll‐like receptor signaling pathways (Figure 3J).

Collectively, these findings demonstrate that CD72^hi^ macrophages exhibit enhanced communication with endothelial cells and promote endothelial transcriptional reprogramming characterized by oxidative stress, inflammatory activation, and pyroptosis‐associated signaling.

### 2.4 CD72^hi^ Macrophages Promote Endothelial Dysfunction and Pyroptotic Activation

**FIGURE 4:**
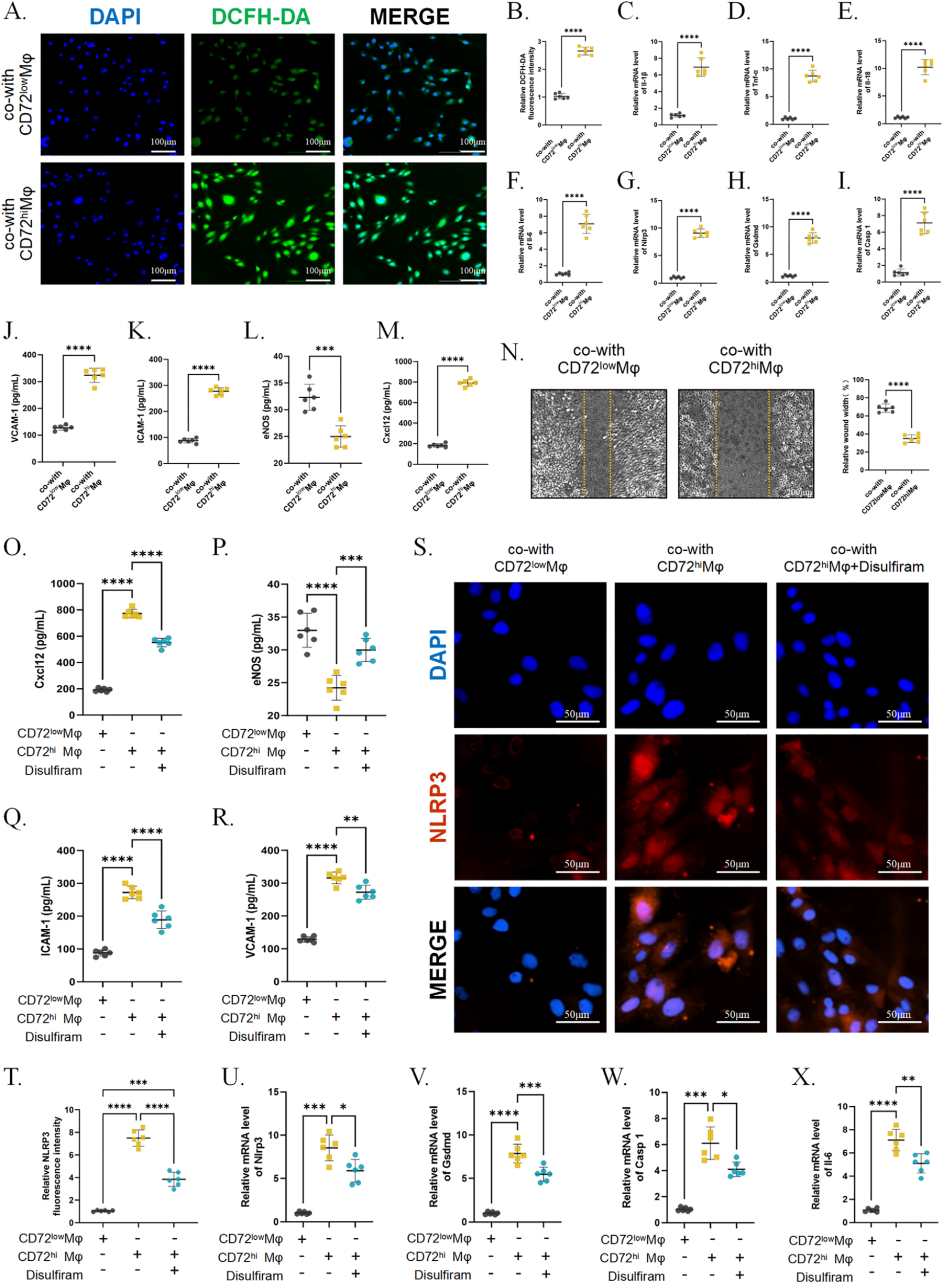
CD72^hi^ macrophages induce oxidative stress, inflammatory activation, and pyroptotic signaling in endothelial cells, and these effects are modulated by pyroptosis inhibition in co‐culture. (A, B) Representative Immunofluorescence images of MAECs co‐cultured with CD72^hi^ or CD72^low^ macrophages and stained with DCFH‐DA (green) and DAPI (blue), together with quantification of relative 2′,7′‐dichlorodihydrofluorescein diacetate (DCFH‐DA) fluorescence intensity (*n* = 6 per group). Scale bars, 100 µm. (C–F) Relative mRNA expression of *Il1b* (C), *Tnf* (D), *Il6* (E), and *Il18* (F) in MAECs after co‐culture with CD72^low^ or CD72^hi^ macrophages (*n* = 6 per group). (G–I) Relative mRNA expression of *Nlrp3* (G), *Gsdmd* (H), and *Casp1* (I) in MAECs after co‐culture with CD72^hi^ or CD72^low^ macrophages (*n* = 6 per group). (J–M) Concentrations of vascular cell adhesion molecule 1 (VCAM‐1) (J), intercellular adhesion molecule 1 (ICAM‐1) (K), endothelial nitric oxide synthase (eNOS) (L), and CXCL12 (M) in co‐culture supernatants from MAECs exposed to CD72^hi^ or CD72^low^ macrophages (*n* = 6 per group). (N) Representative images and quantification of relative wound width in scratch‐wound assays of MAECs after co‐culture with CD72^hi^ or CD72^low^ macrophages (*n* = 6 per group). Scale bars, 200 µm. (O–R) Concentrations of CXCL12 (O), eNOS (P), ICAM‐1 (Q), and VCAM‐1 (R) in co‐culture supernatants from MAECs co‐cultured with CD72^low^ macrophages, with CD72^hi^ macrophages, or with CD72^hi^ macrophages plus disulfiram (*n* = 6 per group). (S–T) Representative immunofluorescence images of MAECs stained for NLRP3 (red) and DAPI (blue), together with quantification of relative NLRP3 fluorescence intensity under the three co‐culture conditions (*n* = 6 per group). Scale bars, 50 µm. (U–X) Relative mRNA expression of *Nlrp3* (U), *Gsdmd* (V), *Casp1* (W), and *Il6* (*X*) in MAECs under the three co‐culture conditions (*n* = 6 per group).

To further evaluate the functional effects of CD72^hi^ macrophages on endothelial cells, we established a transwell co‐culture system using sorted CD72^hi^ or CD72^low^ macrophages and MAECs. ROS generation in MAECs, assessed by 2′,7′‐dichlorodihydrofluorescein diacetate (DCFH‐DA) fluorescence staining, was markedly increased after co‐culture with CD72^hi^ macrophages compared with CD72^low^ macrophages (Figure 4A,B), indicating enhanced endothelial oxidative stress. In parallel, quantitative real‐time polymerase chain reaction (qPCR) analysis revealed that MAECs exposed to CD72^hi^ macrophages expressed higher levels of pro‐inflammatory cytokine transcripts, including *Il1b, Tnf, Il6*, and *Il18*, compared with those co‐cultured with CD72^low^ macrophages (Figure 4C–F). Expression of canonical pyroptosis‐associated genes, including *Nlrp3, Gsdmd*, and *Casp1*, was likewise increased in MAECs co‐cultured with CD72^hi^ macrophages (Figure 4G–I), consistent with activation of endothelial inflammasome–pyroptosis‐related signaling.

We next evaluated endothelial activation and functional impairment induced by CD72^hi^ macrophages. enzyme‐linked immunosorbent assay (ELISA) analysis of cell culture supernatants showed that MAECs co‐cultured with CD72^hi^ macrophages exhibited increased levels of VCAM‐1, ICAM‐1, and CXCL12, accompanied by reduced eNOS levels, compared with those co‐cultured with CD72^low^ macrophages (Figure 4J–M). Furthermore, scratch‐wound assays demonstrated impaired migration capacity of MAECs exposed to CD72^hi^ macrophages, as reflected by delayed wound closure and increased relative wound width compared with the CD72^low^ macrophage co‐culture group (Figure 4N).

To further investigate whether pyroptosis‐associated signaling contributed to CD72^hi^ macrophages‐mediated endothelial dysfunction, we treated the CD72^hi^ macrophages–MAEC co‐culture system with disulfiram, a pharmacological inhibitor of gasdermin D‐mediated pyroptosis. Compared with the CD72^low^ macrophage condition, MAECs co‐cultured with CD72^hi^ macrophages exhibited increased expression of *Nlrp3, Gsdmd, Casp1*, and *Il18*, whereas disulfiram treatment partially reduced the expression of these pyroptosis‐associated genes (Figure 4U–X). Consistently, analysis of culture supernatants showed that disulfiram partially reversed the abnormal endothelial secretory profile induced by CD72^hi^ macrophages, as evidenced by decreased CXCL12, ICAM‐1, and VCAM‐1 levels and partially restored eNOS levels (Figure 4O–R). Moreover, immunofluorescence staining demonstrated increased NLRP3 fluorescence intensity in MAECs following CD72^hi^ macrophage co‐culture, which was attenuated after disulfiram treatment (Figure 4S,T).

Collectively, these data demonstrate that CD72^hi^ macrophages promote endothelial oxidative stress, inflammatory activation, and pyroptosis‐associated signaling, while pharmacological inhibition of pyroptosis‐associated signaling partially alleviates CD72^hi^ macrophages‐induced endothelial dysfunction.

### 2.5 Pharmacological Screening Identifies Ilexoside K as a Candidate Anti‐Atherosclerotic and Anti‐Pyroptotic Small Molecule

**FIGURE 5:**
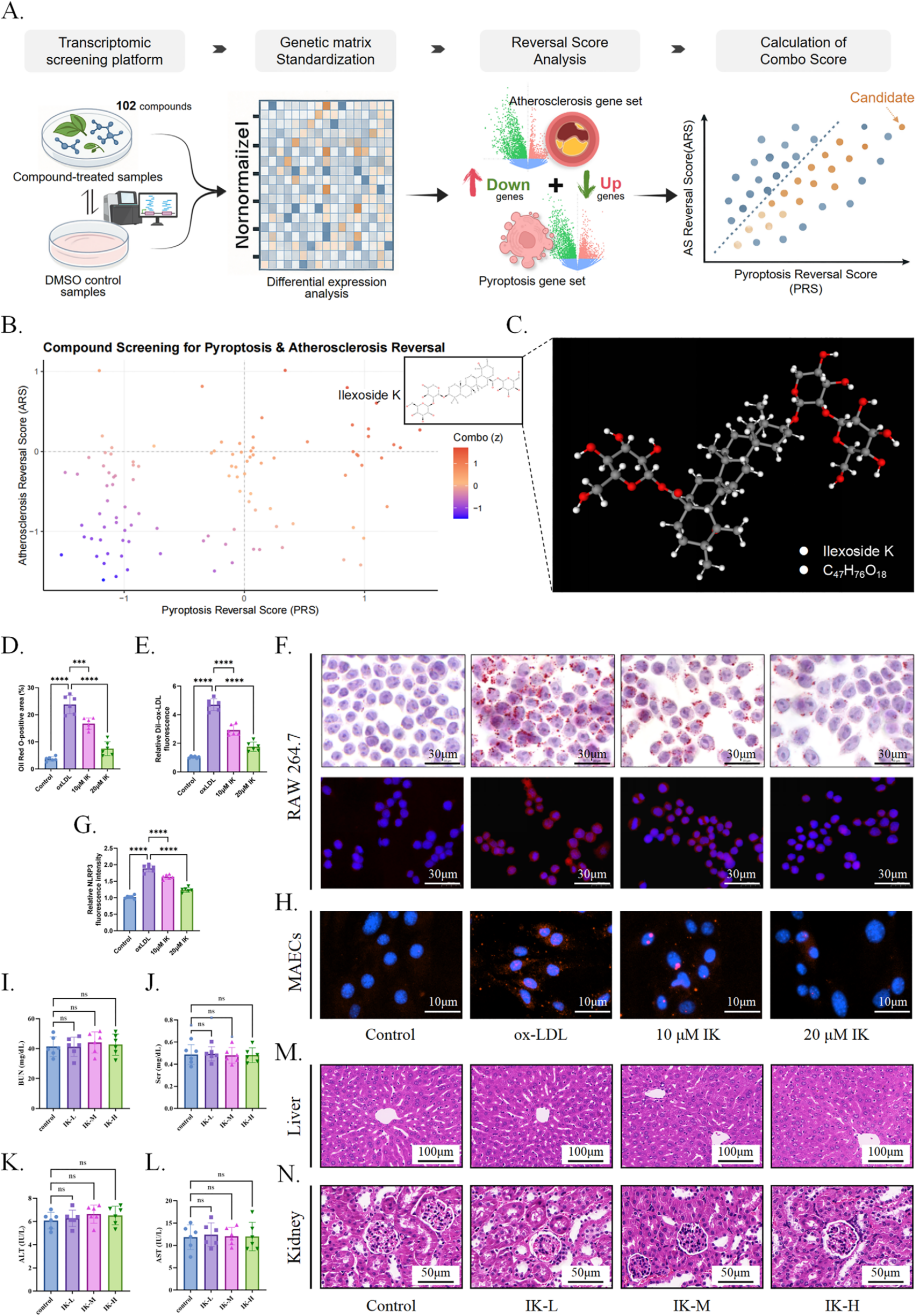
Pharmacological screening identifies Ilexoside K and evaluates its cellular activity and in vivo safety. (A) Schematic diagram of the transcriptome‐based phenotypic screening strategy used to evaluate 102 plant‐derived natural compounds for reversal of pyroptosis‐ and atherosclerosis‐related gene signatures. (B) Scatter plot of atherosclerosis reversal score (ARS) versus pyroptosis reversal score (PRS) for screened compounds, with each dot representing one compound and IK highlighted together with its two‐dimensional chemical structure. (C) Three‐dimensional molecular structure of IK with its chemical formula indicated. (D–F) Quantification of Oil Red O‐positive area (D), quantification of relative Dil‐ox‐LDL fluorescence intensity (E), and representative Oil Red O staining and Dil‐ox‐LDL fluorescence images (F) of RAW264.7 macrophages in the Control, ox‐LDL, 10 µm IK, and 20 µm IK groups (*n* = 6 per group). In (F), the upper panels show Oil Red O staining, and the lower panels show Dil‐ox‐LDL fluorescence (red) with DAPI nuclear counterstaining (blue). Scale bars, 30 µm. (G, H) Quantification of relative NLRP3 fluorescence intensity (G) and representative immunofluorescence images (H) of MAECs in Control, ox‐LDL, 10 µm IK, and 20 µm IK groups (*n* = 6 per group). NLRP3 is shown in red and nuclei are counterstained with DAPI in blue. Scale bars, 10 µm. (I–L) Serum levels of blood urea nitrogen (BUN, I), creatinine (Scr, J), alanine aminotransferase (ALT, K), and aspartate aminotransferase (AST, L) in control, IK‐low, IK‐medium, and IK‐high groups of C57 mice after 28 days of oral administration (*n* = 6 per group). (M, N) Representative H&E‐stained sections of the liver (M) and kidney (N) from mice in the control, IK‐L, IK‐M, and IK‐H groups after 28 days of oral administration. Scale bars, 100 µm in (M) and 50 µm in (N).

To identify small molecules with potential anti‐atherosclerotic and anti‐pyroptotic properties, we established a transcriptome‐based phenotypic screening strategy using a library of 102 plant‐derived natural compounds (Figure 5A). For each compound, differential gene expression profiles relative to dimethyl sulfoxide (DMSO)‐treated controls were generated and systematically compared with curated gene signatures associated with atherosclerosis progression and pyroptotic activation. Based on these analyses, a pyroptosis reversal score (PRS) and an atherosclerosis reversal score (ARS) were calculated for each compound. The integrated PRS and ARS scores were subsequently used to prioritize compounds with potential anti‐pyroptotic and anti‐atherosclerotic activities. Pareto front analysis identified several candidate molecules with favorable reversal profiles, among which IK emerged as a top‐ranked compound based on its combined anti‐pyroptotic and anti‐atherosclerotic signatures (Figure 5B). The chemical structure of IK was further illustrated by two‐ and three‐dimensional structural models (Figure 5C).

We next evaluated the effects of IK on macrophage lipid accumulation under atherosclerosis‐associated stress conditions. In RAW264.7 macrophages, ox‐LDL stimulation markedly increased intracellular lipid accumulation, as demonstrated by Oil Red O staining and Dil‐ox‐LDL fluorescence imaging. Treatment with IK at the selected concentrations of 10 and 20 µm significantly reduced ox‐LDL‐induced lipid accumulation, with a stronger inhibitory effect observed at the higher concentration (Figure 5F). Quantitative analysis confirmed that IK significantly decreased both Oil Red O‐positive lipid area and Dil‐ox‐LDL fluorescence intensity in ox‐LDL‐treated macrophages (Figure 5D,E), indicating that IK suppresses ox‐LDL‐induced macrophage lipid loading. The effects of IK on macrophage viability and the rationale for dose selection were further evaluated by Cell Counting Kit‐8 (CCK‐8) assays, which showed that IK exhibited no obvious cytotoxicity and partially rescued ox‐LDL‐induced reduction in cell viability at the selected concentrations (Figure S3C,D).

We further examined whether IK could regulate endothelial inflammasome‐associated responses under lipid stress conditions. In MAECs, ox‐LDL exposure markedly increased NLRP3 immunofluorescence intensity, whereas treatment with IK at 10 and 20 µm substantially attenuated this increase (Figure 5H). Quantitative analysis confirmed that IK significantly reduced NLRP3 fluorescence intensity in ox‐LDL‐stimulated MAECs (Figure 5G), suggesting that IK attenuates lipid stress‐induced endothelial inflammasome‐associated responses.

Finally, we evaluated the in vivo safety profile of IK administration. Healthy C57 mice were randomly assigned to four groups (control, IK‐low, IK‐medium, and IK‐high) and treated with different doses of IK by oral administration for 28 days. Serum biochemical analysis showed no significant alterations in renal and hepatic function indicators, including blood urea nitrogen (BUN), creatinine (Scr), alanine aminotransferase (ALT), and aspartate aminotransferase (AST), among the different treatment groups (Figure 5I–L). Histological examination of liver and kidney tissues further demonstrated preserved tissue architecture without apparent hepatic or renal pathological abnormalities after IK administration (Figure 5M,N). Together, these findings indicate that IK, identified through a transcriptome‐based screening strategy targeting atherosclerosis‐ and pyroptosis‐associated signatures, exhibits favorable cellular activity and an acceptable short‐term safety profile in vivo.

### 2.6 Ilexoside K Improves Plaque Burden, Dyslipidemia, and Systemic Inflammation in ApoE^−/−^ Mice

**FIGURE 6:**
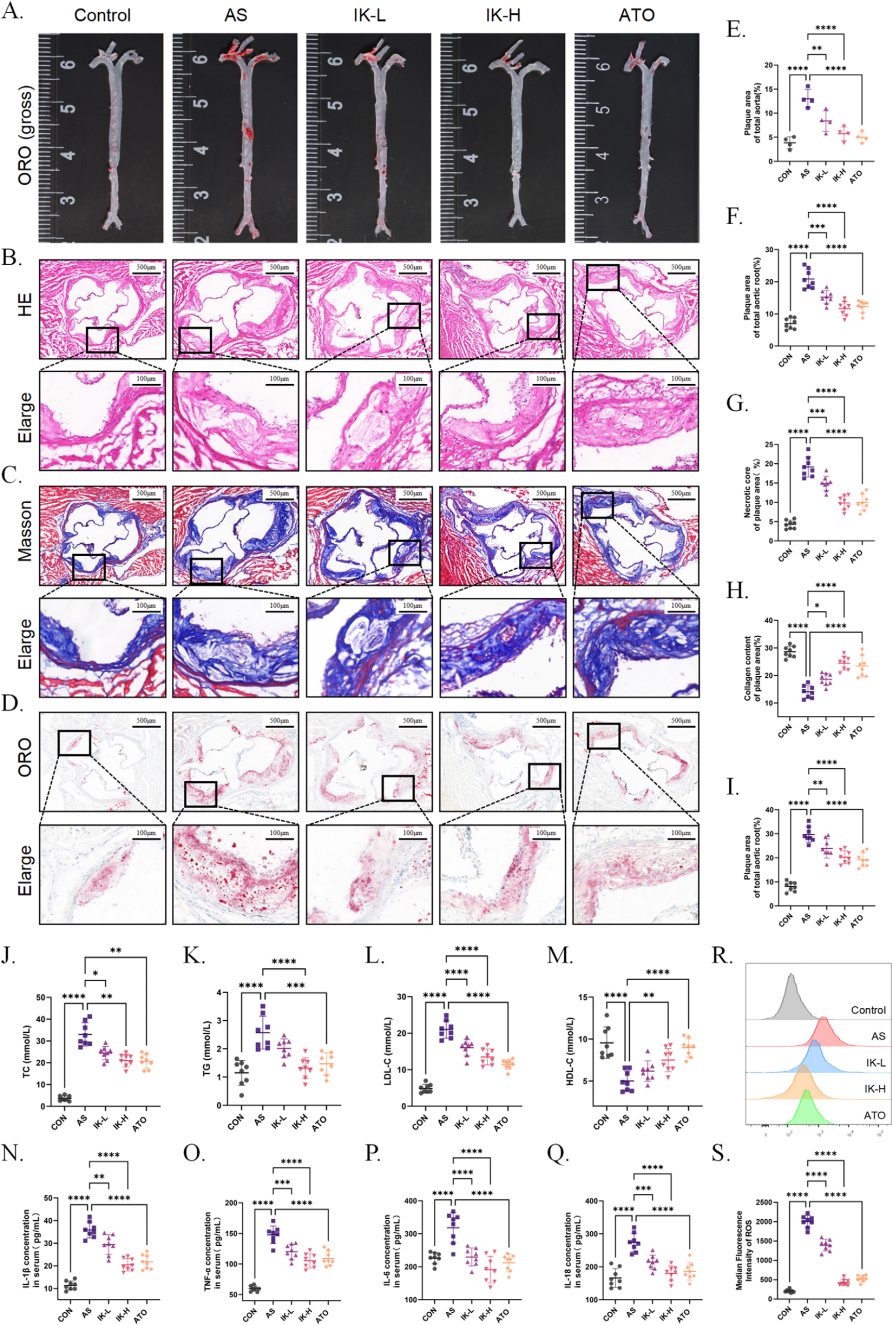
Ilexoside K attenuates atherosclerotic plaque burden, dyslipidemia, systemic inflammation, and vascular oxidative stress in ApoE^−/−^ mice. (A) En‐face Oil Red O staining of whole aortas from the control, atherosclerosis (AS), low‐dose IK‐L, high‐dose IK‐H, and atorvastatin (ATO) groups. (B) Haematoxylin–eosin staining of aortic root sections from five experimental groups with corresponding magnified views. (C) Masson's trichrome staining of aortic root sections from five experimental groups with corresponding magnified views. (D) Oil Red O staining of aortic root sections from five experimental groups with corresponding magnified views. (E) Quantification of Oil Red O–positive area in whole aortas from the five experimental groups (*n* = 4 per group). (F) Quantification of plaque area in H&E–stained aortic root sections from the five experimental groups (*n* = 8 per group). (G) Quantification of necrotic core area in H&E–stained aortic root sections from the five experimental groups (*n* = 8 per group). (H) Quantification of collagen content in Masson's trichrome–stained aortic root sections from the five experimental groups (*n* = 8 per group). (I) Quantification of Oil Red O–positive area in aortic root sections from the five experimental groups (*n* = 8 per group). (J–M) Serum levels of total cholesterol (J), triglycerides (K), LDL‐C (L) and HDL‐C (M) in the five experimental groups (*n* = 8 per group). (N–Q) Serum concentrations of IL‐1β (N), TNF‐α (O), IL‐6 (P) and IL‐18 (Q) in the five experimental groups (*n* = 8 per group). (R, S) Flow cytometry histograms of ROS levels in aortic cell suspensions (R) and quantification of ROS mean fluorescence intensity (S) from five experimental groups (*n* = 8 per group).

To evaluate the therapeutic efficacy of IK in vivo, ApoE‐/‐ mice were subjected to high‐fat diet feeding and randomly assigned to untreated atherosclerosis (AS), low‐dose IK, high‐dose IK, or atorvastatin treatment groups, with control mice maintained under baseline conditions. En‐face Oil Red O staining of whole aortas showed marked lipid deposition in atherosclerotic mice compared with non‐atherosclerotic controls, whereas treatment with low‐dose IK, high‐dose IK, or atorvastatin significantly reduced Oil Red O‐positive lesion area (Figure 6A,E). Consistently, histological analysis of aortic root sections revealed increased plaque area and necrotic core size in the atherosclerotic group and attenuated by IK and reference treatment, with a more pronounced effect at the higher IK dose (Figure 6B,F,G). Masson's trichrome staining demonstrated reduced collagen content within plaques in the untreated atherosclerotic group, while IK and reference treatment increased collagen deposition, again with a stronger effect at the higher IK dose (Figure 6C,H). Oil Red O staining of aortic root sections confirmed the en‐face findings, with reduced lipid‐rich area in IK‐ and reference‐treated mice compared with the untreated atherosclerotic group (Figure 6D,I).

We next assessed whether IK modulated systemic lipid metabolism and inflammatory status. Serum biochemistry showed that TC, TG and LDL‐C were elevated, and HDL‐C was reduced, in atherosclerotic mice relative to controls (Figure 6J–M). Low‐dose IK partially improved TC and LDL‐C, whereas high‐dose IK and reference treatment more broadly reduced TC, TG and LDL‐C while increasing HDL‐C (Figure 6J–M). In parallel, ELISA revealed that circulating IL‐1*β*, TNF‐*α*, IL‐6 and IL‐18 were markedly increased in the atherosclerotic group and diminished by both doses of IK and the reference treatment, with the higher IK dose showing a greater reduction than the lower dose (Figure 6N–Q). These data indicate that IK improves both plaque morphology and systemic cardiometabolic and inflammatory profiles in ApoE^−/−^ mice.

Finally, we investigated the impact of IK on vascular oxidative stress. Flow cytometry of aortic cell suspensions showed higher ROS levels in atherosclerotic mice than in controls, whereas treatment with IK or the reference drug reduced ROS levels, with high‐dose IK producing the most pronounced attenuation (Figure 6R,S). Together, these findings demonstrate that IK alleviates atherosclerotic plaque burden, improves systemic lipid metabolism, dampens systemic inflammation, and mitigates vascular oxidative stress in ApoE^−/−^ mice.

### 2.7 Ilexoside K Suppresses CD72^hi^ Macrophages Accumulation and Alleviates Endothelial Pyroptosis Within Atherosclerotic Plaques

**FIGURE 7:**
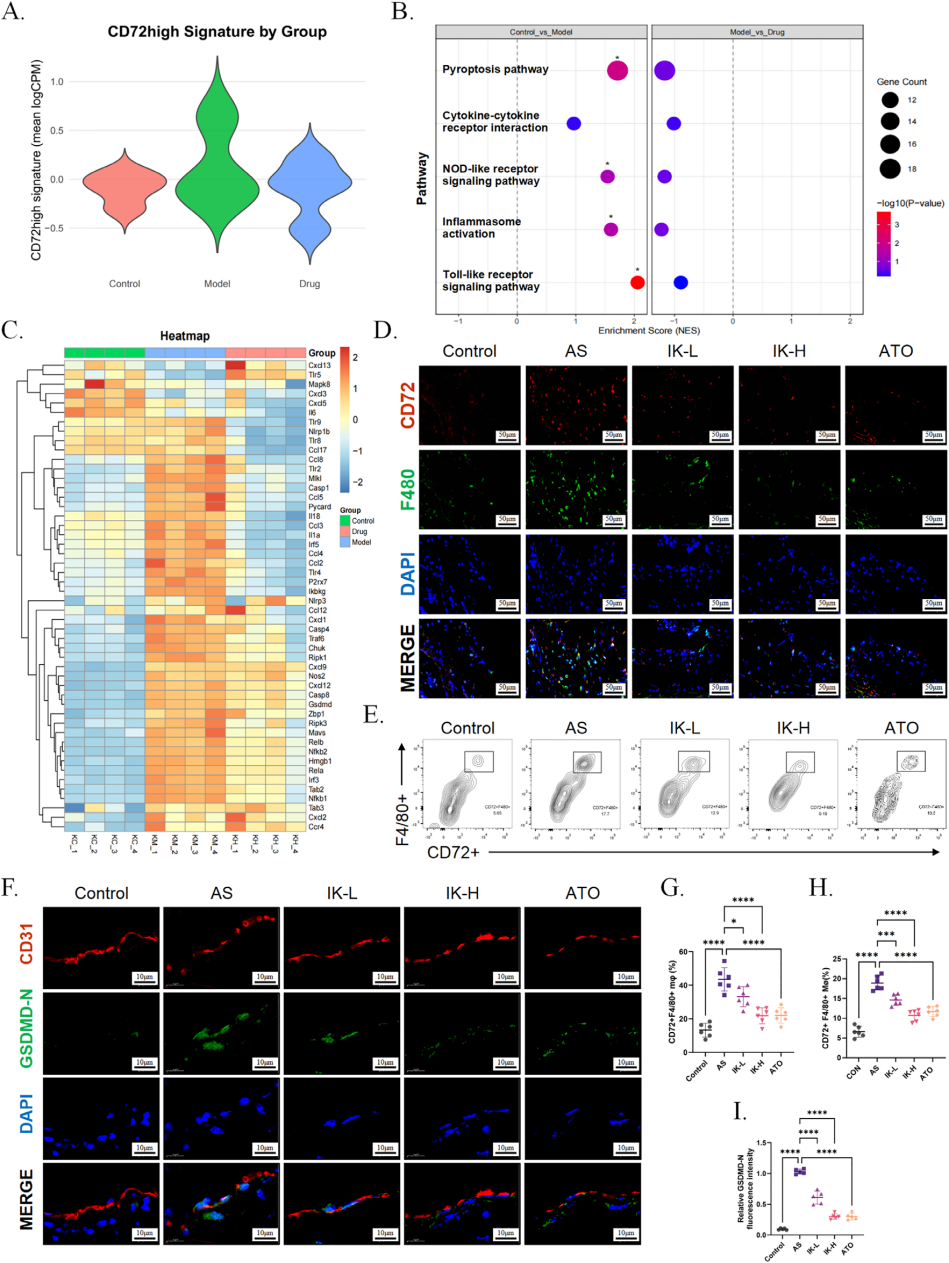
Ilexoside K modulates CD72^hi^ transcriptional signatures, CD72^hi^ macrophages, and endothelial pyroptosis in atherosclerotic aortas. (A) Violin plot of a CD72^hi^ transcriptional signature (mean log CPM) across three experimental groups based on aortic bulk RNA‐seq. (B) Bubble plot of KEGG pathway enrichment for pyroptosis, cytokine–cytokine receptor interaction, NOD‐like receptor signaling, inflammasome activation, and Toll‐like receptor signaling in two pairwise comparisons, with bubble size indicating gene count and color indicating −log10(*p* value). (C) Heatmap of selected genes related to pyroptosis, inflammation, and chemokine signaling across three experimental groups in aortic transcriptomes. (D,G) Representative Immunofluorescence images of aortic root sections for CD72 (red), F4/80 (green), and DAPI (blue) (D), together with quantification of CD72^+^F4/80^+^ macrophages in aortic root lesions (H) from the five experimental groups (*n* = 6 per group). Scale bars, 50 µm. (E,H) Flow cytometry dot plots of aortic cell suspensions showing CD72 and F4/80 expression, together with quantification of CD72^+^F4/80^+^ macrophages in five experimental groups (*n* = 6 per group). (F,I) Representative Immunofluorescence images of aortic root sections for CD31 (red), GSDMD‐N (green), and DAPI (blue), together with quantification of GSDMD‐N fluorescence intensity within CD31^+^ endothelial areas in the five experimental groups (*n* = 5 per group). Scale bars, 10 µm.

To investigate whether the therapeutic effects of IK were associated with regulation of CD72^hi^ macrophages and endothelial pyroptotic activation in vivo, we first analysed aortic bulk RNA‐seq data from control, atherosclerotic, and IK‐treated ApoE^−/−^ mice. A CD72^hi^ macrophage transcriptional signature derived from CD72^hi^ macrophages was calculated based on the mean expression level of signature genes. Compared with control mice, atherosclerotic mice exhibited a marked increase in the CD72^hi^ macrophages signature, whereas IK treatment significantly reduced this signature, indicating suppression of the CD72^hi^ macrophages‐associated transcriptional programme (Figure 7A).

We next performed pathway‐based analysis of differentially expressed genes using KEGG enrichment analysis. In comparison with control mice, atherosclerotic vessels showed activation of pyroptosis‐associated and inflammatory pathways, including pyroptosis, cytokine–cytokine receptor interaction, NOD‐like receptor signaling, inflammasome activation, and Toll‐like receptor signaling pathways. Conversely, these pathways were substantially reversed following IK treatment (Figure 7B). Consistently, heatmap analysis demonstrated coordinated regulation of genes involved in inflammation, inflammasome activation, pyroptosis, and chemokine signaling across the three groups (Figure 7C). To further validate the regulation of CD72^hi^ macrophages by IK at the protein and cellular levels, Western blot analysis of aortic tissues showed that CD72 protein expression was markedly increased in atherosclerotic mice compared with controls, whereas both low‐ and high‐dose IK treatment, as well as atorvastatin treatment, significantly reduced CD72 expression (Figure S3E,F). Consistent with these findings, immunofluorescence staining of aortic root sections demonstrated enhanced CD72 and F4/80 signals and increased CD72 and F4/80 co‐localization within atherosclerotic plaques. In contrast, IK treatment markedly decreased the accumulation of CD72^+^F4/80^+^ macrophages, with a stronger reduction observed in the high‐dose IK group (Figure 7D). Quantitative analysis confirmed a significant decrease in CD72^+^F4/80^+^ macrophage abundance following IK administration (Figure 7G).

Flow cytometric analysis of digested aortic tissues further identified the CD72^+^F4/80^+^ macrophage population across different treatment groups (Figure 7E). Quantification demonstrated that atherosclerotic mice exhibited a marked expansion of CD72^+^F4/80^+^ macrophages, whereas IK and atorvastatin treatment significantly reduced this macrophage subset (Figure 7H), further confirming that IK suppresses CD72^hi^ macrophage accumulation within atherosclerotic lesions. We next evaluated whether IK‐mediated suppression of CD72^hi^ macrophages was accompanied by reduced endothelial pyroptotic activation. Immunofluorescence staining for CD31 and GSDMD‐N in aortic root sections revealed a pronounced increase in CD31^+^GSDMD‐N^+^ endothelial cells in atherosclerotic plaques compared with controls, whereas IK and atorvastatin treatment markedly attenuated GSDMD‐N accumulation within endothelial cells (Figure 7F). Quantitative analysis confirmed a significant reduction in endothelial GSDMD‐N fluorescence intensity after IK treatment (Figure 7I). In addition, CD31/CASP1 double immunofluorescence staining further demonstrated enhanced endothelial caspase‐1 activation in atherosclerotic lesions, which was significantly reduced following IK administration (Figure S3G), providing complementary evidence that IK suppresses endothelial inflammasome–pyroptosis activation.

Collectively, these findings demonstrate that IK inhibits the expansion of CD72^hi^ macrophages within atherosclerotic plaques and alleviates endothelial pyroptotic activation, supporting the role of CD72^hi^ macrophages–mediated endothelial pyroptosis as a therapeutic mediator of IK in atherosclerosis.

### 2.8 Ilexoside K Attenuates CD72^hi^ Macrophages‐Induced Endothelial Pyroptosis and Dysfunction In Vitro

**FIGURE 8:**
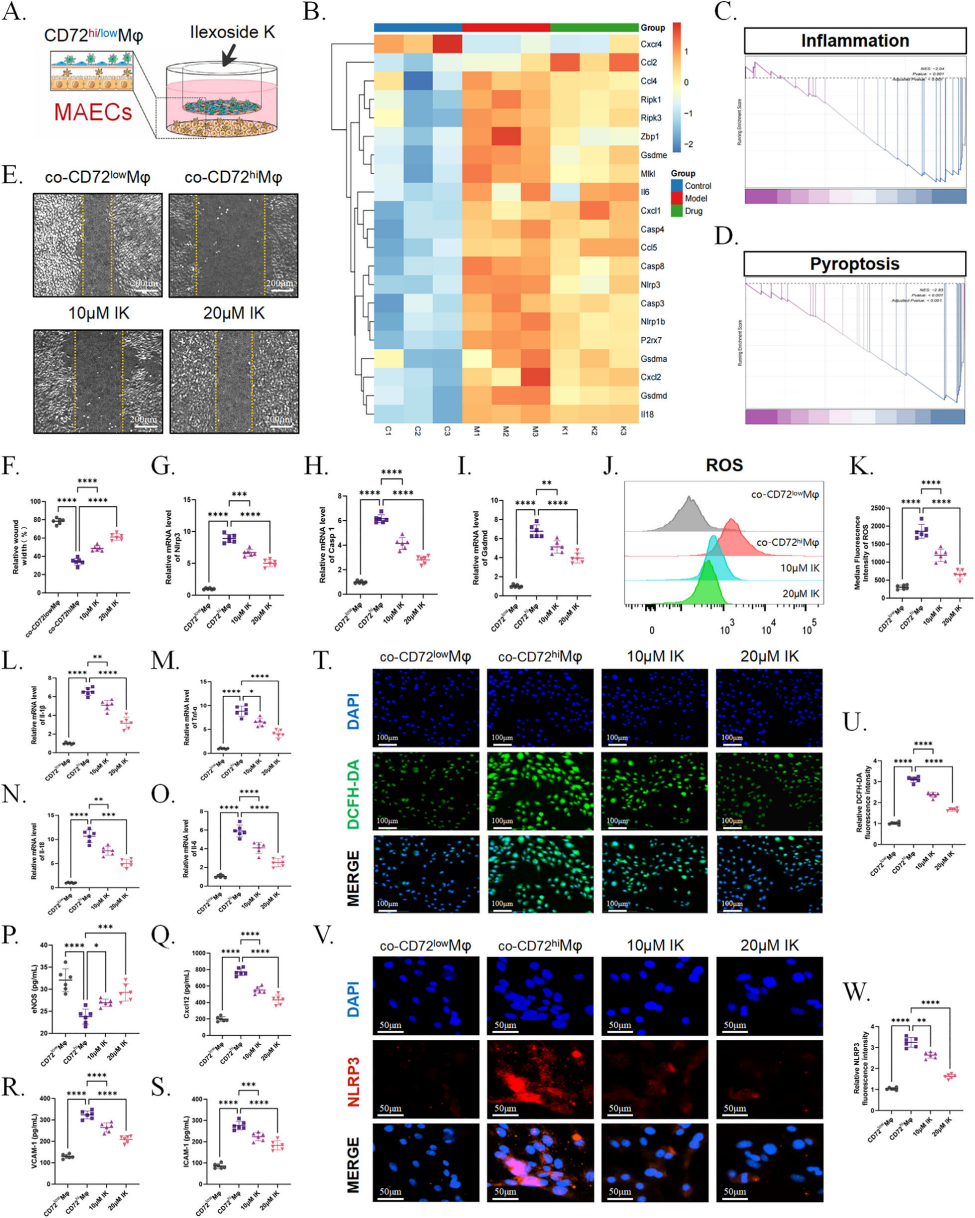
Ilexoside K counteracts CD72^hi^ macrophages–induced endothelial transcriptional reprogramming, pyroptosis, oxidative stress, and dysfunction in co‐culture. (A) Schematic diagram of the transwell co‐culture system in which MAECs are cultured with CD72^hi^ or CD72^low^ macrophages and treated with IK. (B) Heatmap of pyroptosis‐related genes based on RNA‐seq of MAECs under three experimental conditions. (C) GSEA plot of an inflammation‐related gene set comparing two MAEC conditions from the co‐culture system. (D) GSEA plot of a pyroptosis‐related gene set comparing the same two MAEC conditions. (E) Representative images of scratch‐wound assays of MAEC monolayers under four experimental conditions after co‐culture and IK treatment. Scale bars, 200 µm. (F) Bar graph of relative wound width (%) in MAEC scratch assays under four experimental conditions (*n* = 6 per group). (G–I) Relative mRNA expression of *Nlrp3* (G), *Casp1* (H), and *Gsdmd* (I) mRNA expression in MAECs under four experimental conditions (*n* = 6 per group). (J,K) Flow cytometry histograms of ROS levels (J) and quantification of mean fluorescence intensity (K) in MAECs under four experimental conditions (*n* = 6 per group). (L–O) Relative mRNA expression of *Il1b* (L), *Tnf* (M), *Il18* (N), and *Il6* (O) in MAECs under four experimental conditions (*n* = 6 per group). (P–S) Concentrations of eNOS (P), CXCL12 (Q), VCAM‐1 (R), and ICAM‐1 (S) in MAEC culture supernatants under four experimental conditions (*n* = 6 per group). (T,U) Representative Immunofluorescence images of MAECs stained with DCFH‐DA (green) and DAPI (blue) (V) and quantification of relative DCFH‐DA fluorescence intensity (T) under four conditions (*n* = 6 per group). Scale bars, 100 µm. (V,W) Representative Immunofluorescence images of MAECs stained with NLRP3 (green) and DAPI (blue) (W) and quantification of relative NLRP3 fluorescence intensity (V) under four conditions (*n* = 6 per group). Scale bars, 50 µm.

To determine whether IK directly counteracts CD72^hi^ macrophages‐induced endothelial injury, we established a transwell co‐culture system in which MAECs were co‐cultured with sorted CD72^low^ or CD72^hi^ macrophages and subsequently treated with different concentrations of IK (Figure 8A). Bulk RNA sequencing was performed on MAECs from different co‐culture conditions. Heatmap analysis showed that exposure to CD72^hi^ macrophages induced a transcriptional programme characterized by increased expression of pyroptosis‐associated genes, including *Nlrp3, Casp1, Gsdmd*, together with multiple inflammatory mediators, whereas IK treatment partially reversed these transcriptional alterations (Figure 8B). GSEA further revealed that inflammatory and pyroptosis‐related gene signatures were significantly enriched in MAECs co‐cultured with CD72^hi^ macrophages but were attenuated following IK treatment (Figure 8C,D).

To further determine which programmed cell death pathway was preferentially associated with CD72^hi^ macrophages‐mediated endothelial injury, we compared nine major programmed cell death‐related pathways using single‐sample gene set enrichment analysis. Among these pathways, pyroptosis showed the most prominent enrichment pattern in MAECs exposed to CD72^hi^ macrophages and was attenuated after IK treatment, whereas other cell death signatures exhibited relatively modest or inconsistent alterations (Figure S4A). To further evaluate whether necroptosis contributed to the vascular injury phenotype in vivo, CD31/p‐MLKL immunofluorescence staining was performed in both bone marrow transplantation and global Rel‐deficient atherosclerotic models. No significant alteration in endothelial p‐MLKL fluorescence intensity was observed between Rel‐deficient and corresponding control groups (Figure S4B–E), suggesting that necroptotic activation is unlikely to represent a major component of this pathological process.

We next evaluated the functional consequences of CD72^hi^ macrophages–endothelial interaction and the protective effects of IK. Scratch‐wound assays demonstrated that endothelial repair capacity was markedly impaired following CD72^hi^ macrophages co‐culture compared with CD72^low^ macrophages, whereas IK treatment significantly improved wound closure in a concentration‐dependent manner, as shown by representative images and quantitative analysis of relative wound width (Figure 8E,F). At the molecular level, CD72^hi^ macrophages markedly increased the expression of canonical pyroptosis‐related genes, including Nlrp3, Casp1, and Gsdmd, in MAECs, while IK treatment significantly reduced the expression of these genes (Figure 8G–I).

We further investigated oxidative stress responses in endothelial cells. Flow cytometric analysis using DCFH‐DA staining demonstrated that CD72^hi^ macrophages markedly enhanced intracellular ROS accumulation in MAECs compared with CD72^low^ macrophages, whereas IK treatment significantly reduced ROS generation, with a stronger inhibitory effect observed at the higher concentration (Figure 8J,K). Consistently, immunofluorescence staining showed increased DCFH‐DA fluorescence in MAECs exposed to CD72^hi^ macrophages, which was substantially attenuated by IK treatment, and quantitative analysis confirmed that IK significantly decreased endothelial ROS fluorescence intensity (Figure 8T,U).

In parallel, we assessed endothelial inflammatory activation and functional alterations. qPCR analysis showed that CD72^hi^ macrophages increased the expression of inflammatory cytokine transcripts, including Il1b, Tnf, Il6, and Il18, whereas IK treatment markedly suppressed these inflammatory responses (Figure 8L–O). Analysis of endothelial functional markers in culture supernatants revealed that CD72^hi^ macrophages reduced eNOS secretion while increasing CXCL12, VCAM‐1, and ICAM‐1 production. IK treatment partially restored these endothelial functional abnormalities by increasing eNOS levels and reducing CXCL12, VCAM‐1, and ICAM‐1 secretion (Figure 8P–S).

Finally, we examined inflammasome activation at the protein level. Immunofluorescence staining demonstrated enhanced NLRP3 activation in MAECs following CD72^hi^ macrophages exposure, whereas IK treatment substantially reduced NLRP3 fluorescence signals, and quantitative analysis further confirmed that IK significantly decreased NLRP3 fluorescence intensity induced by CD72^hi^ macrophages (Figure 8V,W). Together with the transcriptional and functional findings, these results indicate that IK suppresses CD72^hi^ macrophages‐induced endothelial oxidative stress, inflammatory activation, and pyroptosis‐associated signaling.

Collectively, these findings demonstrate that IK protects endothelial cells from CD72^hi^ macrophages‐mediated injury by attenuating oxidative stress, inflammatory responses, and pyroptosis‐associated activation.

### 2.9 Rel‐Dependent CD72^hi^ Macrophages Reconstitution Attenuates the Anti‐Atherosclerotic Effects of Ilexoside K In Vivo

**FIGURE 9:**
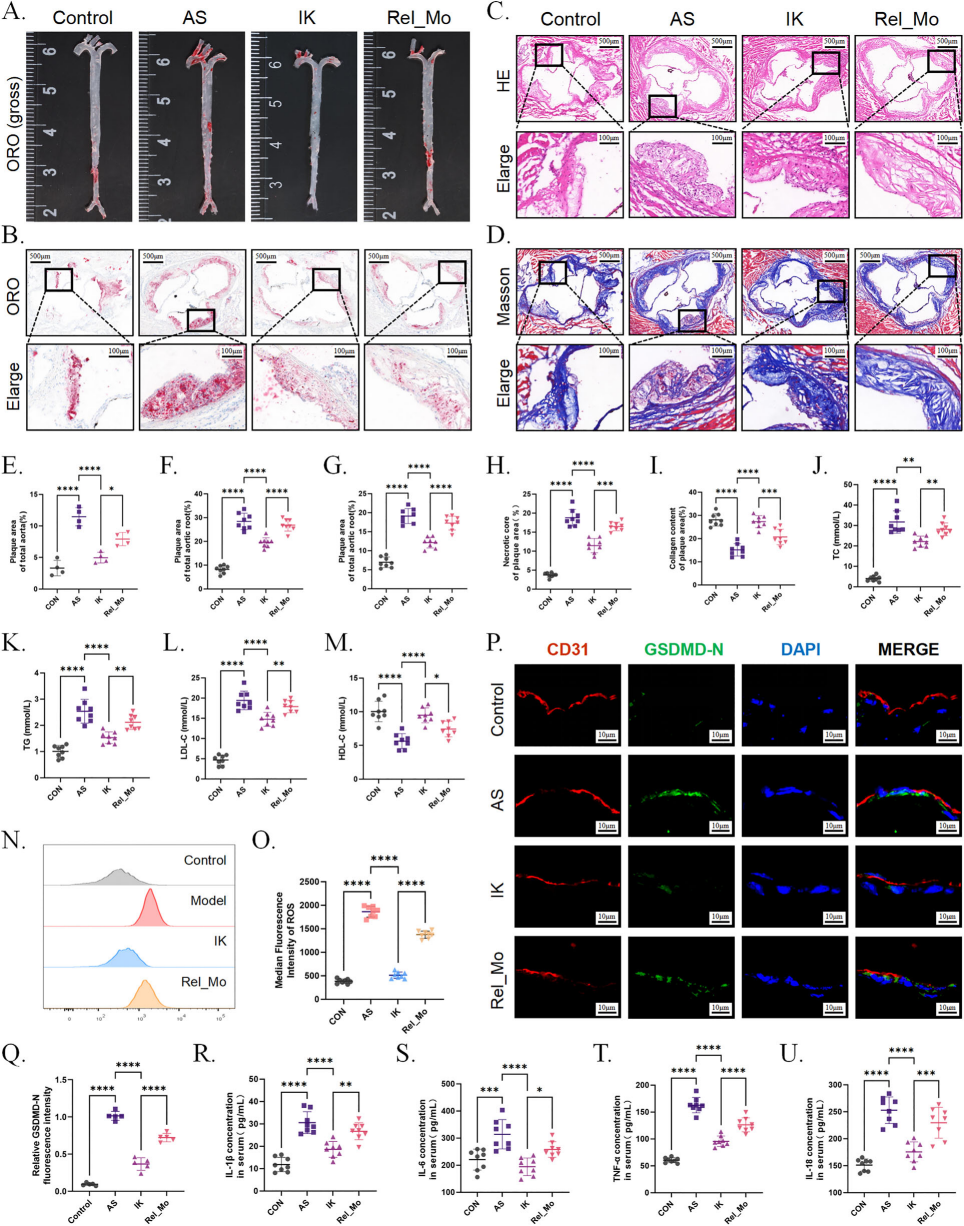
Reconstitution of Rel‐dependent CD72^hi^ macrophages reverses the vascular protective effects of Ilexoside K in ApoE^−/−^ mice. (A) Representative en‐face Oil Red O staining of whole aortas from four experimental groups. (B) Representative Oil Red O staining of aortic root sections from the four experimental groups, with corresponding magnified views. (C) Representative haematoxylin–eosin staining of aortic root sections from four experimental groups with corresponding magnified views. (D) Representative Masson's trichrome staining of aortic root sections from four experimental groups with corresponding magnified views. (E) Quantification of Oil Red O–positive area in whole aortas from four experimental groups (*n = *4 per group). (F) Quantification of Oil Red O–positive area in aortic root sections from four experimental groups (*n = *8 per group). (G) Quantification of necrotic core area on haematoxylin–eosin–stained aortic root sections from four experimental groups (*n = *8 per group). (H) Quantification of plaque area on haematoxylin–eosin–stained aortic root sections from four experimental groups (*n = *8 per group). (I) Quantification of collagen content on Masson's trichrome–stained aortic root sections from four experimental groups (*n = *8 per group). (J–M) Serum levels of total cholesterol (J), triglycerides (K), LDL‐C (L), and HDL‐C (M) levels in four experimental groups (*n = *8 per group). (N, O) Representative flow cytometry histograms showing ROS‐associated fluorescence (N) and quantification of ROS mean fluorescence intensity (O) in aortic cell suspensions from the four experimental groups (*n* = 8 per group). (P, Q) Representative immunofluorescence images of aortic root sections stained for CD31 (red), GSDMD‐N (green), and DAPI (blue), together with quantification of GSDMD‐N fluorescence intensity within CD31^+^ endothelial areas in the four experimental groups (*n* = 6 per group). Scale bars, 10 µm.

To determine whether Rel‐dependent CD72^hi^ macrophages contribute to the vascular protective effects of IK, we performed a reconstitution experiment in ApoE^−/−^ mice, in which Rel‐overexpressing monocytes (Rel_Mo) were intravenously administered during IK treatment. En‐face Oil Red O staining of whole aortas demonstrated that IK markedly reduced lipid deposition compared with untreated atherosclerotic mice, whereas Rel_Mo administration partially reversed this protective effect (Figure 9A). Consistently, histological analysis of aortic root sections using Oil Red O, haematoxylin–eosin, and Masson's trichrome staining showed that IK reduced plaque burden, lipid accumulation, and necrotic core formation while increasing collagen deposition, whereas these plaque‐stabilizing effects were attenuated following Rel_Mo reconstitution (Figure 9B–D).

Quantitative analysis further confirmed that IK significantly decreased whole‐aorta Oil Red O‐positive area, aortic root lipid deposition, plaque area, and necrotic core size, while increasing collagen content. These beneficial effects were partially reversed following Rel_Mo administration (Figure 9E–I). We next evaluated the effects of Rel_Mo reconstitution on systemic lipid metabolism. Serum biochemical analysis showed that IK treatment improved atherosclerosis‐associated dyslipidemia by reducing TC, TG, and LDL‐C levels and increasing HDL‐C levels. In contrast, Rel_Mo administration partially reversed these metabolic improvements, shifting lipid profiles toward the atherosclerotic phenotype (Figure 9J–M). We further assessed vascular oxidative stress by flow cytometry. Representative ROS fluorescence histograms demonstrated elevated ROS accumulation in atherosclerotic mice, which was markedly reduced following IK treatment but restored after Rel_Mo administration (Figure 9N). Quantitative analysis of ROS mean fluorescence intensity confirmed that Rel_Mo reconstitution significantly weakened the inhibitory effect of IK on vascular oxidative stress (Figure 9O).

Finally, we investigated whether Rel‐dependent CD72^hi^ macrophage reconstitution affected endothelial pyroptotic activation within atherosclerotic plaques. Immunofluorescence co‐staining of aortic root sections demonstrated that CD31^+^ endothelial cells in atherosclerotic lesions exhibited markedly increased GSDMD‐N accumulation compared with control mice, whereas IK treatment significantly reduced endothelial GSDMD‐N signals. Notably, Rel_Mo administration partially restored endothelial GSDMD‐N accumulation within plaques, suggesting that Rel‐dependent monocytes promote endothelial pyroptotic activation and counteract the protective effects of IK (Figure 9P). Quantitative analysis confirmed significant differences in endothelial GSDMD‐N fluorescence intensity among the four groups (Figure 9Q).

To further evaluate inflammasome activation upstream of GSDMD‐mediated pyroptosis, CD31/CASP1 double immunofluorescence staining was performed. Compared with control mice, atherosclerotic mice exhibited enhanced endothelial CASP1 activation within plaques, which was markedly suppressed by IK treatment. In contrast, Rel_Mo reconstitution partially restored endothelial CASP1 fluorescence intensity within plaques (Figure S5A), providing complementary evidence that Rel‐dependent CD72^hi^ macrophages contribute to endothelial inflammasome activation and pyroptotic injury. In parallel, circulating inflammatory cytokines, including IL‐1β, IL‐6, TNF‐α, and IL‐18, were elevated in atherosclerotic mice, reduced following IK treatment, and partially restored after Rel_Mo reconstitution (Figure 9R–U).

Collectively, these findings demonstrate that Rel‐dependent monocyte reconstitution partially reverses the protective effects of IK on plaque stabilization, vascular oxidative stress, systemic inflammation, and endothelial pyroptotic activation, supporting an important role for the Rel‐dependent CD72^hi^ macrophages axis in mediating the anti‐atherosclerotic actions of IK.

### 2.10 Ilexoside K Interacts With CXCR4 and Modulates the CD72^hi^ Macrophages–CXCL12/CXCR4 Signaling Axis

**FIGURE 10:**
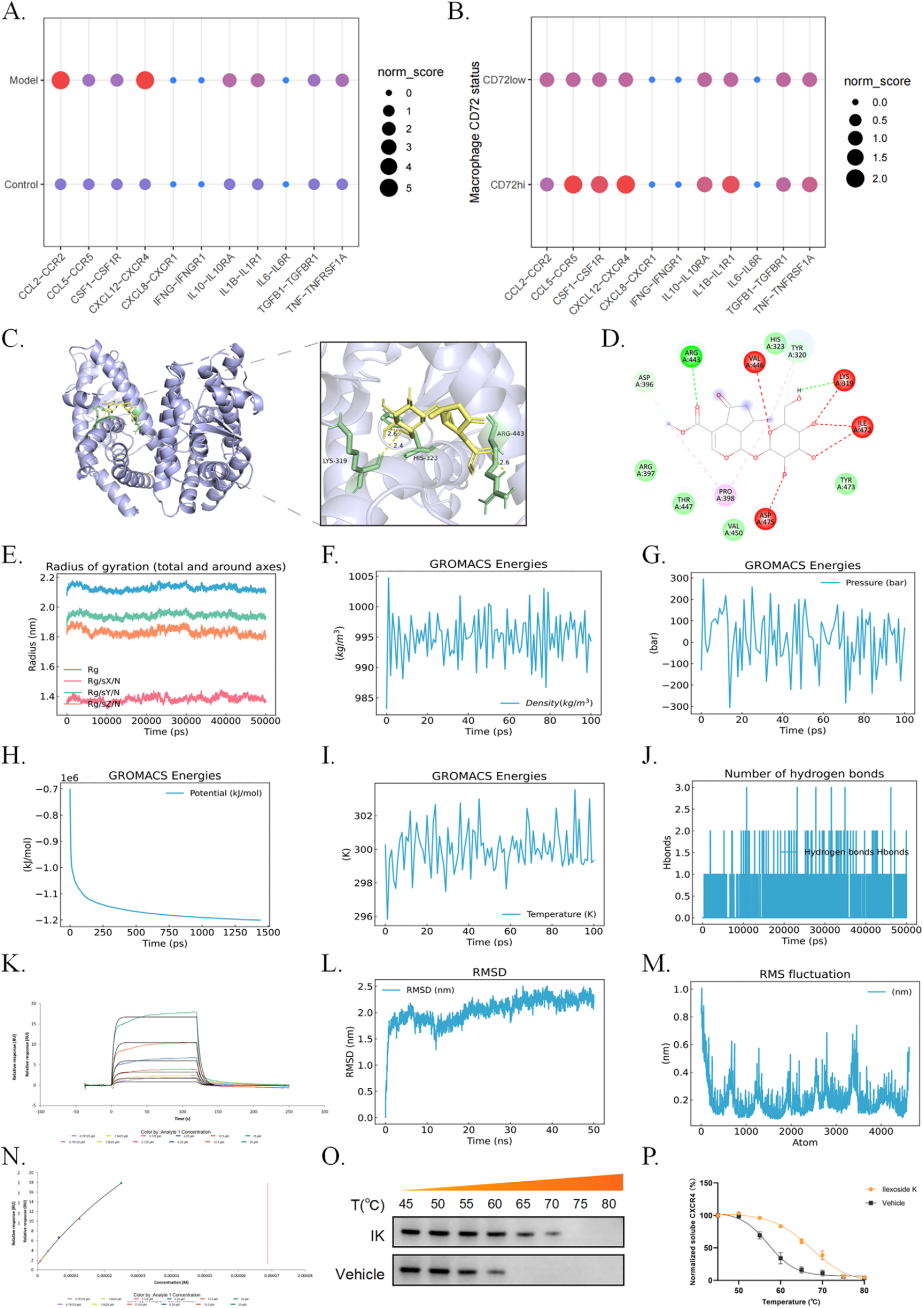
Molecular docking and molecular dynamics simulation of the Ilexoside K–CXCR4 complex. (A) Bubble plot showing selected ligand–receptor interactions under control and atherosclerotic model conditions based on murine aortic single‐cell RNA‐seq data. Bubble size and color represent the normalized interaction scores, as indicated in the legends. (B) Bubble plot comparing selected ligand–receptor interactions involving CD72^low^ and CD72^hi^ macrophages. Bubble size and color represent the normalized interaction scores, as indicated in the legends. (C) Predicted three‐dimensional binding pose of IK within CXCR4. The enlarged view shows the predicted position of IK and its potential interactions with surrounding CXCR4 residues, including Lys319, His323, Arg397, and Arg443. (D) Two‐dimensional interaction map illustrating the predicted hydrogen‐bonding, electrostatic, and hydrophobic contacts between IK and residues within the CXCR4‐binding pocket. (E) Time evolution of the radius of gyration (Rg) of CXCR4 during a 50 ns molecular dynamics simulation, showing the total Rg and Rg along the three principal axes. (F) Time evolution of the simulation‐system density during equilibration. (G) Time evolution of the simulation‐system pressure during equilibration. (H) Potential‐energy profile of the simulation system during energy minimization. (J) Time evolution of the simulation‐system temperature during equilibration. (K) Number of hydrogen bonds formed between IK and CXCR4 during the 50 ns production simulation. (L) Representative SPR sensorgrams showing concentration‐dependent responses of IK to immobilized CXCR4. (M) Root‐mean‐square deviation (RMSD) of CXCR4 backbone atoms over the 50 ns production simulation, indicating structural stability. (M) Root‐mean‐square fluctuation (RMSF) of CXCR4 backbone atoms along the residue index during the production run. (N) Fitted binding curve for the determination of equilibrium dissociation constant (K_D) from SPR analysis. (O) Representative cellular thermal shift assay (CETSA) immunoblots showing CXCR4 abundance in vehicle‐ and IK‐treated cells after heating at the indicated temperatures. (P) Quantification of the CETSA results, showing the relative remaining CXCR4 protein in vehicle‐ and IK‐treated cells across the indicated temperature range. Data were normalized to the signal at the lowest tested temperature.

To further elucidate the molecular mechanism underlying the protective effects of IK, we sought to identify the potential communication pathway linking CD72^hi^ macrophages with endothelial cells. Cell–cell communication analysis based on single‐cell transcriptomic data revealed enhanced predicted interactions between CD72^hi^ macrophages and endothelial cells compared with CD72^low^ macrophages (Figure 10A). Among the predicted ligand–receptor pairs, the CXCL12–CXCR4 axis emerged as a prominent communication pathway, with CXCL12 predominantly associated with macrophages and CXCR4 expressed by endothelial cells (Figure 10B), suggesting that CD72^hi^ macrophages may regulate endothelial dysfunction through CXCL12/CXCR4 signaling.

We next validated the involvement of this signaling axis at the tissue and cellular levels. Immunofluorescence staining of atherosclerotic plaques demonstrated that CD72^+^F4/80^+^ macrophages were spatially associated with enhanced CXCL12 expression, whereas Rel deficiency markedly reduced CD72^+^F4/80^+^ macrophage accumulation and CXCL12 fluorescence intensity within plaques (Figure S6A–C). To determine whether CD72^hi^ macrophages directly contribute to CXCL12 production, CD72^hi^ and CD72^low^ macrophages were isolated and cultured separately. Compared with CD72^low^ macrophages, CD72^hi^ macrophages exhibited significantly increased CXCL12 expression and secretion, as demonstrated by qPCR and ELISA analyses (Figure S6D,E). Furthermore, restoration of Rel‐dependent monocytes (Rel_Mo) in ApoE^−/−^ mice increased CD72^+^F4/80^+^ macrophage accumulation and enhanced CXCL12 expression within atherosclerotic lesions (Figure S6F–H), further supporting the role of Rel‐dependent CD72^hi^ macrophages in regulating CXCL12 production in vivo.

Given the potential involvement of the CXCL12/CXCR4 axis in endothelial injury, we next investigated whether CXCR4 could represent a potential interacting protein involved in the biological activity of IK. Molecular docking analysis using the CXCR4 crystal structure predicted that IK occupied a cavity within the transmembrane domain of CXCR4. IK formed hydrogen bond interactions with key residues, including Lys319, His323, Arg3and Arg443, and established additional polar and hydrophobic contacts with surrounding residues within the binding pocket (Figure 10C,D). These predicted interactions suggested a potential binding mode between IK and CXCR4.

To evaluate the stability of the predicted CXCR4–IK complex, all‐atom molecular dynamics simulations were performed. The radius of gyration analysis showed that CXCR4 maintained overall structural compactness throughout the simulation period (Figure l0E). The simulated system rapidly reached equilibrium after energy minimization and maintained stable density, pressure, and temperature profiles (Figure 10F–I). IK remained associated with CXCR4 during the simulation, maintaining hydrogen bond interactions with the receptor, while RMSD analysis demonstrated that the complex reached a relatively stable conformation after initial equilibration (Figure 10J–L). RMSF analysis further indicated limited flexibility within the transmembrane region containing the predicted binding pocket, supporting the structural stability of the CXCR4–IK interaction (Figure 10M). We subsequently performed experimental validation to examine the interaction between IK and CXCR4. SPR analysis demonstrated concentration‐dependent binding responses between IK and recombinant CXCR4, indicating direct physical interaction between the compound and receptor in vitro (Figure 10N). Furthermore, cellular thermal shift assay (CETSA) showed increased thermal stability of CXCR4 following IK treatment compared with vehicle control, further supporting the interaction between IK and CXCR4 in cells (Figure 10O,P).

Collectively, these findings identify the CXCL12/CXCR4 axis as a potential communication pathway linking CD72^hi^ macrophages to endothelial dysfunction and provide evidence that IK interacts with CXCR4, suggesting that modulation of CXCL12/CXCR4 signaling may contribute to the protective effects of IK against CD72^hi^ macrophages‐mediated endothelial inflammatory and pyroptotic activation.

### 2.11 Endothelial CXCR4 Overexpression Attenuates the Vasculoprotective Effects of Ilexoside K in Atherosclerosis

To determine whether endothelial CXCR4 contributes to the protective effects of IK against atherosclerosis, we performed endothelial CXCR4 overexpression experiments using an adeno‐associated virus (AAV)‐mediated approach. ApoE^−/−^ mice were divided into four groups: AAV‐NC, AAV‐CXCR4, AAV‐NC + IK, and AAV‐CXCR4 + IK. Following IK administration, atherosclerotic lesion development and vascular inflammatory responses were evaluated.

**FIGURE 11:**
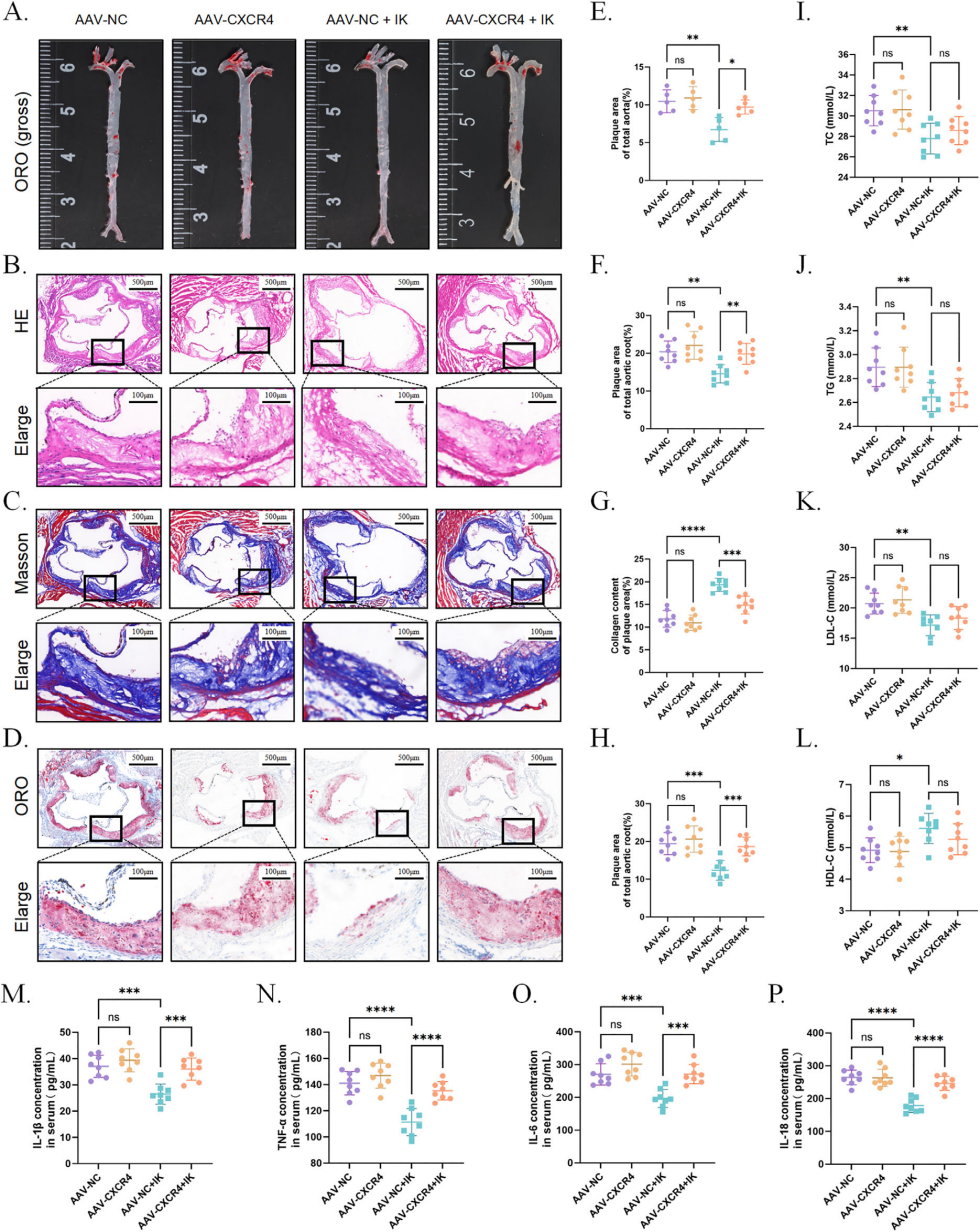
Endothelial CXCR4 overexpression attenuates the vasculoprotective effects of Ilexoside K in atherosclerosis. (A) Representative gross images of aortas stained with Oil Red O from AAV‐NC, AAV‐CXCR4, AAV‐NC + IK, and AAV‐CXCR4 + IK mice. (B) H&E staining of aortic root sections from the four experimental groups, with corresponding magnified views. Scale bars are indicated in the images. (C) Masson staining of aortic root sections with magnified views showing plaque collagen content. Scale bars are indicated in the images. (D) Oil Red O staining of aortic root sections with magnified views indicating intraplaque lipid accumulation. (E) Quantification of Oil Red O‐positive lesion area in whole aortas from the four experimental groups (*n* = 5 per group). (F) Quantification of plaque area in H&E‐stained aortic root sections (*n* = 8 per group). (G) Quantification of collagen content in Masson's trichrome‐stained aortic root sections (*n* = 8 per group). (H) Quantification of Oil Red O‐positive lesion area in aortic root sections (*n* = 8 per group). (I–L) Serum levels of total cholesterol (I), triglycerides (J), LDL cholesterol (K), and HDL cholesterol (L) in the four experimental groups (*n* = 8 per group). (M–P) Serum concentrations of IL‐1β (M), TNF‐α (N), IL‐6 (O), and IL‐18 (P) in the four experimental groups (*n* = 8 per group).

En‐face Oil Red O staining of whole aortas showed that IK treatment markedly reduced lipid deposition in AAV‐NC mice, whereas endothelial CXCR4 overexpression partially diminished this protective effect, resulting in increased aortic lipid accumulation (Figure 11A,E). Consistently, histological analysis of aortic root sections by haematoxylin–eosin staining demonstrated that IK significantly reduced plaque area, while CXCR4 overexpression partially reversed this inhibitory effect on plaque formation (Figure 11B,F). Plaque composition was further assessed by Masson's trichrome and Oil Red O staining. IK treatment increased collagen deposition and reduced lipid‐rich plaque areas, indicating enhanced plaque stability. However, endothelial CXCR4 overexpression partially abolished these plaque‐stabilizing effects, as evidenced by decreased collagen content and increased lipid accumulation within lesions (Figure 11C,D,G,H). We next examined whether endothelial CXCR4 overexpression influenced systemic lipid metabolism. Serum biochemical analysis showed that IK treatment improved atherosclerosis‐associated dyslipidemia, characterized by reductions in TC, TG, and LDL‐C, together with increased HDL‐C levels. Importantly, CXCR4 overexpression had limited influence on the lipid‐modifying effects of IK, suggesting that endothelial CXCR4 contributes primarily to vascular protection rather than systemic lipid regulation (Figure 11I–L). Given the established role of CXCR4 signaling in vascular inflammatory responses, we further evaluated circulating inflammatory cytokines. IK treatment markedly reduced serum levels of IL‐1β, TNF‐α, IL‐6, and IL‐18 in AAV‐NC mice, whereas endothelial CXCR4 overexpression partially reversed these anti‐inflammatory effects, leading to increased inflammatory cytokine levels (Figure 11M–P).

Collectively, these results demonstrate that endothelial CXCR4 overexpression partially reverses the anti‐atherosclerotic, plaque‐stabilizing, and anti‐inflammatory effects of IK without altering its systemic lipid‐regulatory activity. These findings further support the involvement of CXCR4‐dependent endothelial responses in mediating the vascular protective effects of IK.

### 2.12 Endothelial CXCR4 Overexpression Compromises the Endothelial‐Protective Effects of Ilexoside K

Following the observation that endothelial CXCR4 overexpression attenuated the anti‐atherosclerotic effects of IK in vivo, we next investigated the underlying cellular mechanisms responsible for this phenotype. Specifically, we examined whether CXCR4 overexpression altered IK‐mediated regulation of endothelial injury, oxidative stress, macrophage responses, and pyroptotic activation within atherosclerotic lesions.

**FIGURE 12:**
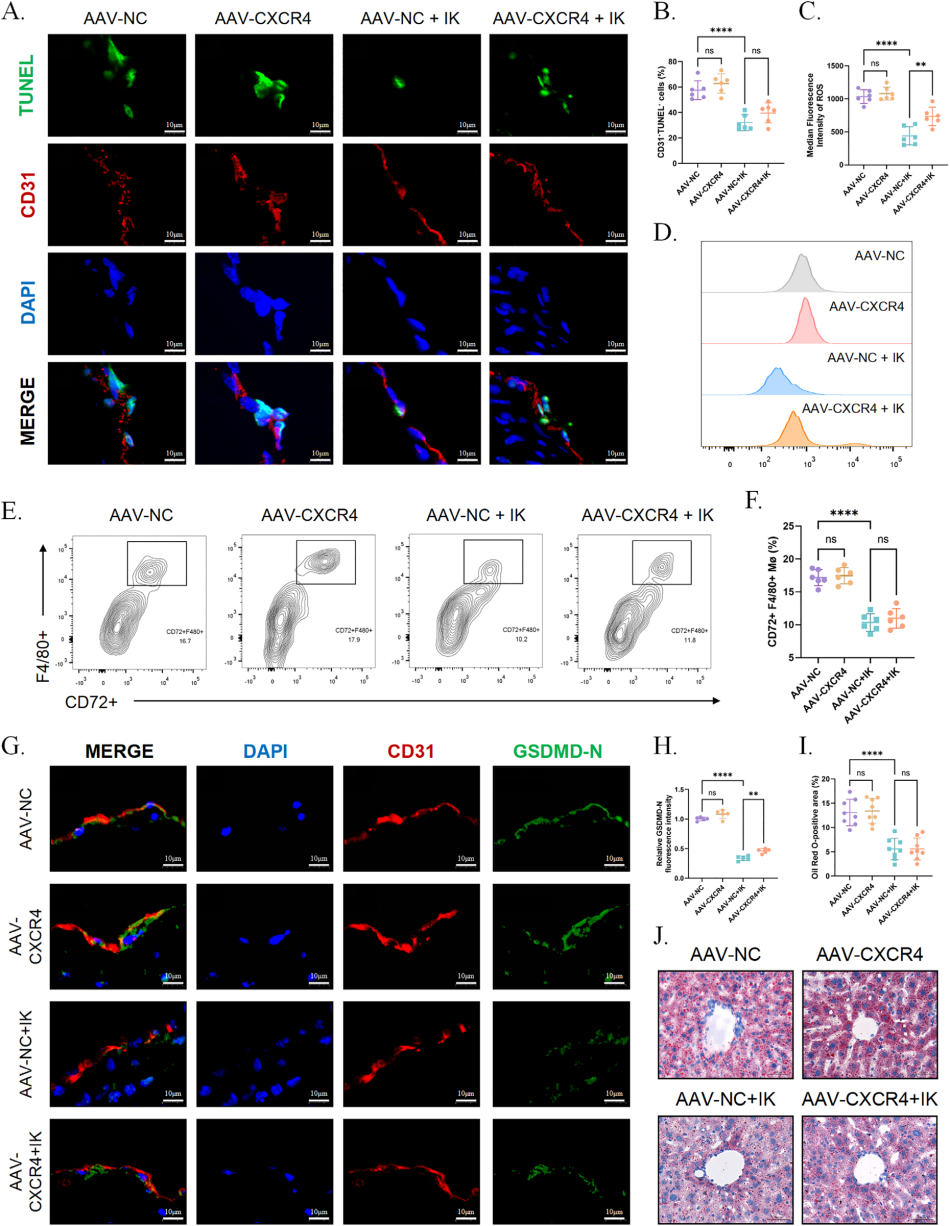
Endothelial CXCR4 overexpression compromises the anti‐inflammatory and endothelial‐protective effects of Ilexoside K. (A, B) Representative immunofluorescence images of aortic root sections stained for TUNEL (green), CD31 (red), and DAPI (blue), together with quantification of TUNEL‐positive endothelial cells in the AAV‐NC, AAV‐CXCR4, AAV‐NC + IK, and AAV‐CXCR4 + IK groups (*n* = 6 per group). Scale bars, 10 µm. (C, D) Quantification of ROS‐associated fluorescence intensity in aortic single‐cell suspensions (C) and representative flow cytometry histograms (D) in the four experimental groups (*n* = 6 per group). (E, F) Representative flow cytometry plots showing CD72 and F4/80 expression in aortic cell suspensions (E), together with quantification of CD72^+^F4/80^+^ macrophages (F) in the four experimental groups (*n* = 6 per group). (G,H) Representative immunofluorescence images of aortic root sections stained for CD31 (red), GSDMD‐N (green), and DAPI (blue) (G), together with quantification of GSDMD‐N fluorescence intensity within CD31^+^ endothelial areas (H) in the four experimental groups (*n* = 5 per group). (I, J) Quantification of hepatic Oil Red O‐positive area (I) and representative Oil Red O‐stained liver sections (J) from the four experimental groups (*n* = 8 per group). Scale bars, 50 µm.

We first assessed endothelial injury‐associated changes within atherosclerotic plaques. CD31/TUNEL immunofluorescence staining of aortic root sections showed that IK treatment markedly reduced TUNEL‐positive signals within CD31^+^ endothelial regions in AAV‐NC mice, whereas endothelial CXCR4 overexpression partially restored endothelial TUNEL positivity under IK treatment (Figure 12A,B). These findings suggest that excessive endothelial CXCR4 expression weakens the protective effect of IK against vascular endothelial injury. Vascular oxidative stress was subsequently evaluated by flow cytometry using DCFH‐DA staining of aortic single‐cell suspensions. Representative fluorescence profiles and quantitative analysis demonstrated that IK significantly reduced ROS accumulation in AAV‐NC mice, whereas endothelial CXCR4 overexpression partially abolished this inhibitory effect, resulting in increased ROS levels despite IK treatment (Figure 12C,D). These data indicate that CXCR4 overexpression compromises the antioxidative effects of IK within the vascular microenvironment.

We next investigated whether endothelial CXCR4 overexpression influenced the abundance of CD72^hi^ macrophages. Flow cytometric analysis of aortic single‐cell suspensions showed that IK markedly reduced the proportion of CD72^+^F4/80^+^ macrophages, whereas this reduction was not significantly restored by endothelial CXCR4 overexpression (Figure 12E,F). Consistently, Western blot analysis of aortic tissues demonstrated that CXCR4 overexpression did not substantially alter aortic CD72 protein abundance under IK treatment (Figure S7A,B). These results suggest that endothelial CXCR4 primarily regulates downstream endothelial responses rather than affecting the accumulation of CD72^hi^ macrophages. We further examined endothelial pyroptosis‐associated responses within plaques. CD31/GSDMD‐N immunofluorescence staining revealed that IK markedly reduced GSDMD‐N accumulation in CD31^+^ endothelial cells, whereas endothelial CXCR4 overexpression partially restored endothelial GSDMD‐N signals (Figure 12G,H). In parallel, immunofluorescence staining for CD31/CASP1 and CD31/NLRP3 showed that IK suppressed inflammasome‐associated signaling in endothelial cells, while CXCR4 overexpression partially reversed these effects (Figure S7C–F). Together, these findings indicate that excessive endothelial CXCR4 expression attenuates the inhibitory effects of IK on endothelial inflammasome‐associated pyroptotic responses.

To exclude the possibility that CXCR4‐mediated effects were secondary to alterations in systemic lipid metabolism, hepatic lipid accumulation and cholesterol metabolism were further examined. Oil Red O staining of liver sections showed no obvious exacerbation of hepatic lipid deposition following endothelial CXCR4 overexpression under IK treatment (Figure 12I). Consistently, hepatic TC and triglyceride contents were not significantly altered by CXCR4 overexpression, and the expression of cholesterol metabolism‐related genes, including *Ldlr, Pcsk9, Hmgcr*, and *Srebf2*, showed no substantial reversal of IK‐induced metabolic regulation (Figure S7G–L). These findings suggest that the effects of endothelial CXCR4 overexpression on vascular injury and pyroptosis are largely independent of systemic lipid metabolic changes.

Collectively, these results demonstrate that endothelial CXCR4 overexpression weakens the anti‐inflammatory, antioxidative, and anti‐pyroptotic effects of IK without restoring CD72^hi^ macrophage accumulation or substantially altering systemic lipid metabolism. These findings support a model in which IK alleviates CD72^hi^ macrophages‐associated endothelial injury through modulation of CXCL12–CXCR4‐dependent endothelial responses, thereby limiting inflammasome‐associated pyroptotic activation and vascular lesion progression.

### 2.13 CXCR4‐Dependent Endothelial Protection by Ilexoside K Against CD72^hi^ Macrophages‐Induced Injury In Vitro

**FIGURE 13:**
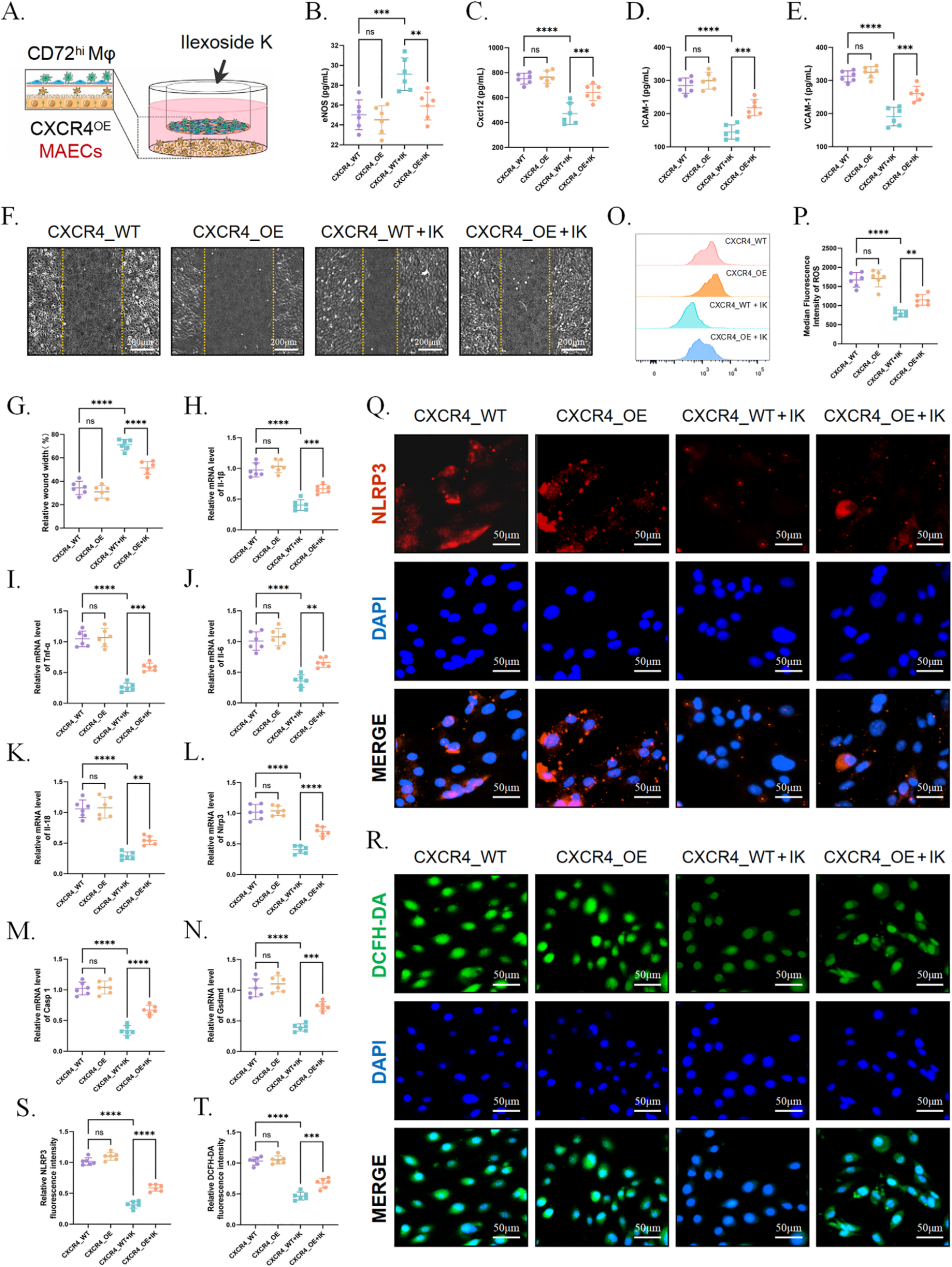
Endothelial CXCR4 overexpression abrogates the protective effects of Ilexoside K against CD72hi macrophages‐induced endothelial injury, oxidative stress, and pyroptosis in vitro. (A) Schematic of the CD72^hi^ macrophages and MAECs transwell co‐culture system. MAECs were transfected with CXCR4 overexpression lentivirus (CXCR4_OE) or control lentivirus (CXCR4_WT) and treated with IK as indicated. (B–E) Concentrations of eNOS (B), CXCL12 (C), ICAM‐1 (D), and VCAM‐1 (E) in co‐culture supernatants from the CXCR4_WT, CXCR4_OE, CXCR4_WT + IK, and CXCR4_OE + IK groups (*n = *6 per group). (F,G) Representative images of scratch‐wound assays (F) and quantification of wound closure (G) in MAECs under the four experimental conditions (*n* = 6 per group). Scale bars, 200 µm. (H–N) Relative mRNA expression of *Il1b* (H), *Tnf* (I), *Il6* (J), *Il18* (K), *Nlrp3* (L), *Casp1* (M), and *Gsdmd* (N) in MAECs under the four experimental conditions (*n* = 6 per group). (O,P) Representative flow cytometry histograms showing ROS‐associated fluorescence (O) and quantification of mean fluorescence intensity (P) in MAECs under the four experimental conditions (*n* = 6 per group). (Q,S) Representative immunofluorescence images of MAECs stained for NLRP3 (red) and DAPI (blue) (Q), together with quantification of relative NLRP3 fluorescence intensity (S), under the four experimental conditions (*n = *6 per group). Scale bars, 50 µm. (R,T) Representative fluorescence images of MAECs stained with DCFH‐DA (green) and DAPI (blue) (R), together with quantification of relative DCFH‐DA fluorescence intensity (T), under the four experimental conditions (*n* = 6 per group). Scale bars, 50 µm.

Having demonstrated that endothelial CXCR4 contributes to the vascular protective effects of IK in vivo, we further investigated whether CXCR4 is involved in the endothelial protective effects of IK using a CD72^hi^ macrophages–MAEC co‐culture model. MAECs were transfected with either CXCR4 overexpression vector (CXCR4_OE) or control vector (CXCR4_WT), followed by co‐culture with sorted CD72^hi^ macrophages in the presence or absence of IK treatment (Figure 13A).

We first examined endothelial functional alterations induced by CD72^hi^ macrophages and the modulatory effects of IK. ELISA analysis of culture supernatants demonstrated that IK partially restored endothelial functional alterations in CXCR4_WT MAECs, characterized by increased eNOS levels and reduced VCAM‐1 and ICAM‐1 secretion. In addition, IK modulated CXCL12‐associated endothelial responses. However, CXCR4 overexpression weakened these regulatory effects of IK (Figure 13B–E). Scratch wound healing assays further showed that CD72^hi^ macrophages impaired endothelial migratory capacity, whereas IK treatment significantly promoted wound closure in CXCR4_WT MAECs. In contrast, CXCR4 overexpression partially attenuated the restorative effect of IK on endothelial migration (Figure 13F,G). We next evaluated oxidative stress responses. Flow cytometric analysis using DCFH‐DA staining demonstrated that IK markedly reduced ROS accumulation induced by CD72^hi^ macrophages in CXCR4_WT MAECs. However, CXCR4 overexpression weakened the inhibitory effect of IK on ROS generation (Figure 13O,P). Consistently, DCFH‐DA immunofluorescence staining confirmed that IK reduced intracellular ROS fluorescence intensity, whereas this antioxidant effect was partially impaired following CXCR4 overexpression (Figure 13R,T). We further investigated inflammatory and pyroptosis‐associated transcriptional responses regulated by endothelial CXCR4. Quantitative real‐time PCR analysis showed that CD72^hi^ macrophage co‐culture markedly increased the expression of inflammatory cytokines, including Il1b, Tnf, Il6, and Il18, in MAECs, whereas IK treatment significantly suppressed these inflammatory responses in CXCR4_WT cells. CXCR4 overexpression partially weakened the inhibitory effects of IK on inflammatory gene expression (Figure 13H–K). In parallel, IK treatment significantly reduced the expression of pyroptosis‐associated genes, including Nlrp3, Casp1, and Gsdmd, in CXCR4_WT MAECs exposed to CD72^hi^ macrophages. CXCR4 overexpression weakened these inhibitory effects and restored the expression of pyroptosis‐associated genes despite IK treatment (Figure 13L–N).

Collectively, these findings demonstrate that endothelial CXCR4 contributes to the protective effects of IK against CD72^hi^ macrophages‐induced endothelial dysfunction, oxidative stress, and inflammasome‐associated pyroptotic responses. CXCR4 overexpression partially counteracts the beneficial effects of IK, providing further cellular evidence supporting the involvement of CXCL12–CXCR4 signaling in the endothelial protective mechanism of IK.

## 3 Discussion

Macrophages‐driven chronic vascular inflammation and endothelial barrier dysfunction are core pathological drivers of ASCVD and the residual cardiovascular risk that persists despite optimal lipid‐lowering therapy [22]. While recent advances in single‐cell transcriptomics have uncovered extensive functional heterogeneity of plaque macrophages, the disease‐relevant pro‐atherogenic subsets that directly drive endothelial injury and their tractable therapeutic targets remain incompletely defined [39, 40]. Pyroptosis, a pro‐inflammatory form of regulated cell death, has emerged as a critical mediator of endothelial damage in atherosclerosis [28], yet the upstream intercellular regulatory mechanisms governing endothelial pyroptosis in the plaque microenvironment remain elusive, and targeted interventions with translational potential are still lacking. In this study, we address these critical unmet clinical and scientific needs with three key advances: first, we identify CD72^hi^ macrophages as a novel c‐Rel‐driven pro‐atherogenic subset enriched in human and murine atherosclerotic lesions, which drives endothelial pyroptosis via pathological crosstalk with vascular endothelial cells; second, we identify the CXCL12–CXCR4 axis as an important signaling pathway involved in CD72hi macrophage–endothelial pathogenic crosstalk; third, we discover and validate IK as a CXCR4‐modulating natural lead compound with dual anti‐atherosclerotic and anti‐pyroptotic activities, which modulates CXCL12–CXCR4 signaling‐associated immune–vascular crosstalk and alleviates atherosclerosis in vivo.

Our study provides the first functional and mechanistic characterization of CD72^hi^ macrophages in atherosclerosis, substantially expanding the current landscape of disease‐relevant macrophage subsets in the atherosclerotic vascular niche. Previous single‐cell studies have identified multiple macrophage populations in atherosclerotic lesions [23, 24], including Trem2^hi^ foam cells [41], Ly6C^hi^ inflammatory macrophages [42], and Lyve1^+^ resident‐like macrophages [23], each with distinct transcriptional features and inferred functional roles. However, few studies have defined macrophage subsets that directly drive endothelial injury during plaque progression—the initiating and sustaining event of atherogenesis. CD72 was originally recognized as a B‐cell co‐receptor [43, 44], with emerging evidence indicating its expression in myeloid cells under inflammatory conditions [45]. Our prior work [25] identified a Rel‐dependent CD72^hi^ macrophage population with robust pro‐inflammatory and tissue‐injurious activity in the injured heart, and here we extend these findings to atherosclerosis, demonstrating that this subset is markedly expanded in both murine and human atherosclerotic plaques, with its abundance positively correlated with plaque burden in mice and coronary atherosclerotic severity in patients. Notably, genetic ablation of Rel‐dependent CD72^hi^ macrophages significantly reduced atherosclerotic lesion formation, systemic inflammation, and endothelial pyroptosis in vivo, establishing a causal role of this subset in atherogenesis. Although Rel is expressed in multiple cell types, our bone marrow transplantation and monocyte reconstitution studies support the involvement of hematopoietic Rel‐dependent CD72^hi^ macrophages in endothelial pyroptosis and atherosclerosis. Future macrophage‐specific Rel deletion studies will be required to establish its cell‐intrinsic role. Functionally, CD72^hi^ macrophages exhibit enhanced intercellular communication with endothelial cells compared with their CD72^low^ counterparts, and directly drive endothelial oxidative stress, inflammasome activation, pyroptotic injury, and dysfunction in co‐culture systems. These findings not only define CD72^hi^ macrophages as a key pathogenic driver of endothelial injury in atherosclerosis, but also provide a novel cellular target for mitigating the inflammatory residual risk of ASCVD.

Our findings further unravel a previously unrecognized mechanism whereby CD72^hi^ macrophages drive endothelial pyroptosis via the CXCL12–CXCR4 signaling axis, filling a critical gap in the understanding of intercellular regulation of endothelial pyroptosis in atherosclerosis. Accumulating evidence has established that endothelial pyroptosis mediated by the NLRP3–caspase‐1–GSDMD axis promotes endothelial dysfunction, accelerates plaque progression, and destabilizes atherosclerotic lesions [29, 46]. However, most existing therapeutic strategies targeting pyroptosis focus on directly inhibiting the core pyroptotic machinery, which may lead to off‐target effects due to the broad physiological roles of these molecules in innate immunity, and lack cell‐type specificity for the vascular endothelium [31, 32]. In this context, targeting the upstream drivers of endothelial pyroptosis in the plaque microenvironment represents a more specific and safer therapeutic strategy. Here, we show that CD72^hi^ macrophages are the key upstream cellular driver of endothelial pyroptosis in atherosclerosis, with the CXCL12–CXCR4 axis acting as a critical signaling pathway involved in this pathogenic macrophage–endothelial crosstalk. Cell‐cell communication analysis based on single‐cell transcriptomics revealed that the CXCL12–CXCR4 axis exhibited the strongest interaction between CD72^hi^ macrophages and endothelial cells in atherosclerotic lesions. More importantly, we demonstrated that endothelial‐specific overexpression of CXCR4 substantially weakened the protective effects of IK against endothelial pyroptosis and atherosclerosis in vivo, without affecting the abundance of CD72^hi^ macrophages in plaques. These findings support the CXCL12–CXCR4 axis as an important downstream signalling pathway mediating CD72^hi^ macrophage‐associated endothelial injury, and provide a mechanistic basis for targeted intervention of endothelial pyroptosis by blocking this immune‐vascular crosstalk. Our findings support CD72^hi^ macrophages as an important CXCL12‐producing population within the plaque microenvironment. However, given the complexity of atherosclerotic lesions, contributions from other CXCL12‐expressing vascular or stromal cells cannot be excluded and warrant further investigation.

Leveraging a transcriptome‐based phenotypic screening platform [36, 37, 38], we identified IK as a top lead compound with dual anti‐atherosclerotic and anti‐pyroptotic activities, and further investigated CXCR4 as a potential molecular mediator of IK activity. Natural products have long been an invaluable source for cardiovascular drug discovery [33, 34], and saponins from *Ilex pubescens* have been reported to exhibit hypocholesterolemic and anti‐inflammatory activities [47, 48]. However, the specific bioactive constituent responsible for its anti‐atherosclerotic effects, its direct molecular target, and the underlying mechanism have remained unclear until now. Our study is the first to demonstrate that IK exerts robust vasculoprotective effects in atherosclerotic mice, including reducing plaque burden, ameliorating dyslipidemia and systemic inflammation, suppressing lesional CD72^hi^ macrophage accumulation, and alleviating endothelial pyroptosis, with a favorable short‐term safety profile in vivo. Mechanistically, we provide multiple lines of evidence supporting an interaction between IK and CXCR4, including molecular docking, all‐atom molecular dynamics simulations, SPR, and CETSA assays. Critically, gain‐of‐function assays demonstrated that endothelial‐specific overexpression of CXCR4 substantially weakened the vascular protective effects of IK both in vitro and in vivo, supporting CXCR4 as a functional mediator of IK‐induced anti‐atherosclerotic and anti‐pyroptotic responses. Notably, reconstitution of Rel‐dependent CD72^hi^ macrophages also abrogated the therapeutic effects of IK in vivo, further supporting that IK acts primarily by interrupting CD72^hi^ macrophages‐CXCR4‐mediated endothelial injury. These findings not only elucidate the pharmacological mechanism of IK for the first time, but also provide a promising natural lead compound for the development of CXCR4‐targeted therapies for ASCVD. Although docking, molecular dynamics simulations, SPR, CETSA, and CXCR4 gain‐of‐function experiments collectively support an interaction between IK and CXCR4, the precise binding residues responsible for this interaction remain to be determined. Future studies using site‐directed mutagenesis and structural validation will be necessary to further define the molecular basis of IK‐associated CXCR4 modulation.

Collectively, our study integrates single‐cell transcriptomics, genetic depletion and gain‐of‐function models, transcriptome‐based drug screening, and molecular target validation to establish a complete mechanistic axis from the pathogenic macrophage subset to endothelial pyroptosis and atherosclerotic progression, and further identifies a targeted therapeutic lead compound. The significance of our work is threefold. First, we define CD72^hi^ macrophages as a novel pro‐atherogenic subset that directly drives endothelial injury, which advances our understanding of macrophage heterogeneity in atherosclerosis and provides a novel cellular biomarker and therapeutic target for ASCVD. Second, we unravel that the CXCL12–CXCR4 axis is an important mediator of the pathological crosstalk between CD72^hi^ macrophages and endothelial cells, which reveals a previously unrecognized upstream regulatory mechanism of endothelial pyroptosis and offers a specific interventional node for vascular protection. Third, we discover and validate IK as a direct CXCR4‐modulating natural compound with dual anti‐atherosclerotic and anti‐pyroptotic activities, which provides a promising translational candidate for the treatment of ASCVD, particularly for addressing the residual inflammatory risk that cannot be managed by current lipid‐lowering therapies.

Several limitations of this study should be acknowledged. First, the pharmacokinetic properties of IK, including its metabolic stability, oral bioavailability, and in vivo half‐life, have not been comprehensively characterized, which are essential for further preclinical development. Second, while our study focuses on endothelial CXCR4 as an important mediator of IK‐induced vascular protection, we cannot exclude potential effects of IK on CXCR4 signaling in other cell populations within the plaque microenvironment, including smooth muscle cells and immune cells. Cell‐specific genetic models will be required to further define the contribution of CXCR4 signaling across different vascular compartments. Third, the downstream signaling events linking CXCR4 inhibition by IK to the suppression of endothelial pyroptosis remain to be fully elucidated. Although available human datasets and experimental analyses support the relevance of CD72^hi^ macrophages and CXCL12–CXCR4 signaling, direct spatial validation in human plaques remains lacking. Future studies using larger human cohorts and spatial profiling approaches will be important to establish the translational relevance of this macrophage–endothelial axis.

In summary, this study systematically elucidates the mechanism by which IK alleviates atherosclerosis through modulating the CXCL12–CXCR4 axis to inhibit CD72^hi^ macrophage‐mediated endothelial pyroptosis. We define CD72^hi^ macrophages as a key driver of atherogenesis via CXCL12–CXCR4‐mediated endothelial pyroptosis, and demonstrate that IK, as a CXCR4‐modulating natural lead compound, effectively alleviates atherosclerosis by interrupting this pathogenic immune‐vascular crosstalk. These findings not only provide novel insights into the immunopathological mechanisms of atherosclerosis, but also open new avenues for the development of targeted therapies for ASCVD, particularly for addressing the residual inflammatory risk that cannot be managed by current lipid‐lowering therapies.

## 4 Experimental Section

### 4.1 Drugs

Ilexoside K (IK; B28007; purity ≥95%) was purchased from Shanghai Yuanye Bio‐Technology Co., Ltd. (Shanghai, China). Atorvastatin calcium tablets were purchased from Pfizer (Pfizer Inc.). Disulfiram (HY‐B0240R; purity 99.54%) was purchased from MedChemExpress (MCE, China).

### 4.2 Mice

ApoE^−/−^ mice (8‐week‐old males, 20 ± 2 g) were purchased from Beijing Vital River Laboratory Animal Technology Co., Ltd. Rel^−/−^ApoE^−/−^ mice were generated by Guangzhou Mingxun Biotechnology Co., Ltd. Mice were housed under a 12 h light–dark cycle at 25°C and 50%–70% relative humidity. All experimental procedures were approved by the Animal Ethics Committee of Guangzhou University of Chinese Medicine (Approval No. 20241220007). Mice were fed either a standard chow diet (Control; MD12016, Medicience Ltd., Yangzhou, China; 4.3% fat, 19.2% protein, 67.3% carbohydrate) or a high‐fat diet (Cat. no. XTHF45, XieTong Pharmaceutical Bio‐Engineering, Nanjing, China; 45% fat, 20% protein, 35% carbohydrate). ApoE^−/−^ mice were fed a high‐fat diet for 8 weeks to induce early‐stage atherosclerosis, as this duration has been widely reported to produce reproducible plaque formation with prominent lipid accumulation and inflammatory macrophage infiltration while avoiding excessive late‐stage plaque complications. To evaluate the effects of IK on atherosclerosis, mice on a high‐fat diet received daily oral gavage of 0.9% saline, IK (12.5 or 25 mg/kg/day), or Atorvastatin (ATO; Lipitor; 20 mg/kg/day) for 8 weeks. To achieve endothelial‐specific overexpression of CXCR4, a recombinant adeno‐associated virus (AAV) carrying the murine CXCR4 coding sequence under the control of the endothelial‐specific VE‐cadherin promoter (AAV‐CXCR4) was constructed. A control virus carrying only the empty vector (AAV‐NC) was generated in parallel. Viral titers were determined by quantitative PCR. Mice received a single intravenous injection of AAV (1 × 10^11^ viral genomes per mouse) via the tail vein two weeks prior to initiating a high‐fat diet and IK treatment.

### 4.3 Preparation of Aortic Single‐Cell Suspensions

Fresh specimens were obtained from the aortas of ApoE^−/−^ mice. Tissues were enzymatically digested using a combination of collagenase, hyaluronidase, and DNase I to generate single‐cell suspensions, followed by filtration through a 40 µm cell strainer and removal of dead cells.

### 4.4 Flow Cytometry

The aorta was dissected as previously described and digested into a cell suspension. The cells were incubated at room temperature in the dark for 60 min with a mixture containing anti‐mouse CD45‐PE (clone 30‐F11, BioLegend), anti‐mouse CD11b‐FITC, and anti‐mouse F4/80‐APC (clone BM8, Invitrogen). Intracellular ROS levels were assessed using the fluorescent probe 2′,7′‐dichlorodihydrofluorescein diacetate (DCFH‐DA). Cells were incubated with DCFH‐DA (10 µm) at 37°C for 60 min in the dark, followed by washing with phosphate‐buffered saline (PBS). DAPI solution (BioLegend) was added to the cell suspension, and the sample was analysed using the BD Biosciences LSR Fortessa high‐parameter flow cytometer. The fluorescence intensity of CD72 and DCF was quantified, and the percentage of CD72hi macrophages and their respective ROS levels were compared across experimental groups.

### 4.5 Isolation and Culture of Primary MAECs

Primary MAECs were isolated using an aortic explant outgrowth method with minor modifications. Briefly, mice were euthanized according to institutional guidelines, and thoracic aortas were aseptically harvested and immediately transferred into ice‐cold sterile PBS containing 1% penicillin–streptomycin. Under a stereomicroscope, residual blood, perivascular adipose tissue, and adventitia were carefully removed to minimize contamination from fibroblasts and vascular smooth muscle cells. The cleaned aortas were opened longitudinally, cut into small segments (1–2 mm), and rinsed repeatedly with PBS. Culture plates were pre‐coated with 0.1% gelatin at 37°C for 30–60 min. Aortic segments were placed luminal side down onto the coated surface and allowed to attach before addition of a minimal volume of endothelial growth medium. After endothelial outgrowth, cells were maintained under endothelial‐selective culture conditions, and low‐passage MAECs were collected for subsequent experiments. The purity and identity of isolated MAECs were validated by flow cytometry before functional assays. Cells were stained with endothelial markers CD31 and VE‐cadherin, together with α‐SMA and F4/80 as exclusion markers for vascular smooth muscle cells and macrophages, respectively.

### 4.6 Flow Cytometric Sorting

Single‐cell suspensions were prepared from mouse aortas as described above. Cells were resuspended in cold staining buffer (PBS supplemented with 2% FBS) and kept on ice. For Fc receptor blockade, cells were incubated with an anti‐mouse CD16/32 antibody (clone 2.4G2; Purified Anti‐Mouse CD16/32 Antibody [2.4G2], Cat. No. E‐AB‐F0997A, Elabscience, Wuhan, China) for 10–15 min at 4°C. Cells were then stained with fluorochrome‐conjugated antibodies at 4°C for 20–30 min in the dark, including CD45‐PE (clone 30‐F11, BioLegend), CD11b‐APC/Fire 750 (clone M1/70, BioLegend), F4/80‐APC (clone BM8, Invitrogen), and CD72‐FITC (clone 10.1.D2, Invitrogen). After staining, cells were washed twice with staining buffer and resuspended in cold sorting buffer. Immediately prior to sorting, DAPI (Cat. No. 422801, BioLegend) was added to identify and exclude dead cells. Fluorescence‐minus‐one (FMO) controls were used to define gating boundaries for CD72. Cell sorting was performed on a MoFlo Astrios EQ cell sorter (Beckman Coulter, Indianapolis, IN, USA).

For flow cytometric quantification of F4/80^+^CD72^+^ macrophages, aortic single‐cell suspensions were stained using the same antibody panel described above. After exclusion of debris, doublets, and DAPI‐positive dead cells, leukocytes were first gated as CD45^+^ cells, followed by identification of macrophages as CD11b^+^F4/80^+^ cells. The proportion of CD72^+^ cells within the F4/80^+^ macrophage population was then quantified. Data were acquired using a flow cytometer and analysed with FlowJo software.

### 4.7 Cell Culture

The complete endothelial growth medium consisted of DMEM/F12 supplemented with 10–20% fetal bovine serum (FBS), endothelial cell growth supplement (ECGS; 50–100 µg/mL), heparin (50–100 U/mL), and 1% penicillin–streptomycin. Cultures were maintained at 37°C in a humidified incubator with 5% CO_2_. Medium was replaced every 2 days. When the outgrowth reached ∼70–80% confluence, cells were harvested by brief incubation with 0.05% trypsin‐EDTA, re‐seeded onto freshly gelatin‐coated plates for expansion. MAECs at early passages (P1–P3) were used for downstream experiments.

Flow‐sorted CD72^hi^ and CD72^low^ macrophages were collected into pre‐chilled tubes containing complete medium (DMEM/F12 supplemented with FBS and ECGS) and were immediately used for subsequent transwell co‐culture experiments with MAECs. For macrophage foam cell induction, flow‐sorted CD72^hi^ and CD72^low^ macrophages were seeded in complete medium and allowed to adhere. The cells were then stimulated with oxidized low‐density lipoprotein (ox‐LDL, 50 µg/mL) for 24 h to establish a foam cell model.

### 4.8 Cell Viability Assay

Cell viability was assessed using a Cell Counting Kit‐8 (CCK‐8) assay. Briefly, cells were seeded in 96‐well plates and treated as indicated. CCK‐8 reagent was then added to each well and incubated at 37°C for 1–2 h. Absorbance was measured at 450 nm using a microplate reader. Cell viability was calculated relative to the control group after blank subtraction.

### 4.9 Cell Co‐Culture

MAECs and flow‐sorted CD72^hi^ or CD72^low^ macrophages were indirectly co‐cultured using a Transwell system equipped with a 0.4‐µm pore‐size polycarbonate membrane. MAECs (1.5 × 10^5^ cells per well) were seeded in the lower chamber of six‐well plates, while CD72^hi^ or CD72^low^ macrophages were seeded in the upper inserts at a comparable density. To mimic a hyperlipidemic environment, ox‐LDL (50 µg/mL) was added to the culture medium during co‐culture.

### 4.10 Endothelial Scratch‐Wound Migration Assay

Endothelial cell migration was assessed using a scratch‐wound assay. MAECs were seeded in 6‐well plates and cultured to a confluent monolayer (90–100%). Cells were then serum‐starved in DMEM/F12 for 6 h. A uniform linear scratch was created across the cell monolayer using a sterile 200‐µL pipette tip. Detached cells were removed by gently washing twice with PBS, and fresh medium was added according to the experimental design. For co‐culture conditions, MAECs in the lower chamber were subjected to transwell co‐culture with flow‐sorted CD72^hi^ or CD72^low^ macrophages in the upper inserts; ox‐LDL (50 µg/mL) was added to the upper chamber and co‐cultured for 24 h as described above. For drug intervention, treatments were applied immediately after scratching and maintained throughout the assay.

Wound closure was documented by capturing images at 0 and 24 h using an inverted microscope under identical fields. The wound area (or wound width) was quantified using ImageJ.

### 4.11 Immunofluorescence Staining

MAECs were seeded onto sterile glass coverslips in 24‐well plates and cultured to the desired confluence. After the indicated treatments, cells were washed twice with PBS, fixed with 4% paraformaldehyde for 15 min at room temperature, and permeabilized with 0.1% Triton X‐100 in PBS for 10 min. Following PBS washes, non‐specific binding was blocked with 5% BSA in PBS for 1 h at room temperature.

Cells were incubated overnight at 4°C with primary antibody pairs anti‐CD31 antibody alongside either anti‐CASP1 or anti‐NLRP3 antibody (Affinity Biosciences LTD; Cat. No. DF7438). After three washes with PBS, cells were incubated for 1 h at room temperature in the dark with an Alexa Fluor 594–conjugated anti‐rabbit IgG (H+L) F(ab′)_2_ fragment secondary antibody (Cell Signaling Technology, Inc.; Cat. No. 8889S) diluted 1:1000. Nuclei were counterstained with DAPI (BioLegend; Cat. No. 422801) for 5 min. Coverslips were washed and mounted using an anti‐fade fluorescence mounting medium (Servicebio, Wuhan, China; Cat. No. G1401). Images were acquired under identical exposure settings using a fluorescence microscope, and NLRP3 fluorescence intensity was quantified using ImageJ when required.

### 4.12 Oxidative Stress Assessment

Intracellular ROS levels were measured using 2′,7′‐dichlorofluorescein diacetate (DCFH‐DA; TargetMol, Cat. No. T15458). According to the experimental design, DCFH‐DA staining was applied for fluorescence imaging in endothelial cells and for quantitative flow‐cytometric analysis in endothelial cell suspensions and single‐cell suspensions prepared from aortic tissues. For adherent endothelial cells, culture medium was removed after the indicated treatments, and cells were incubated with 100 µL DCFH‐DA working solution for 5–30 min at room temperature in the dark. Cells were then washed 2–3 times with culture medium (5 min per wash), counterstained with DAPI (BioLegend, Cat. No. 422801), and fluorescence images were acquired for subsequent quantification. For flow cytometry, endothelial cells were harvested by enzymatic detachment, or aortic tissues were enzymatically digested and filtered to obtain single‐cell suspensions. Cells were washed with PBS, adjusted to approximately 1 × 10^6^ cells/mL, and incubated with 1 mL DCFH‐DA working solution for 5–30 min at room temperature in the dark. Cells were centrifuged at 400 × g for 3–4 min, washed twice with PBS (5 min per wash), and resuspended in serum‐free medium or PBS. DAPI was added immediately prior to acquisition to exclude dead cells; analyses were performed on DAPI‐negative events, and ROS levels were expressed as the mean fluorescence intensity (MFI) of 2′,7′‐dichlorofluorescein (DCF).

### 4.13 Participants and Inclusion Criteria

A total of 20 participants were enrolled, including 10 patients with atherosclerosis (AS) and 10 non‐AS controls. All participants underwent coronary angiography for clinical indications. The AS group was defined by the presence of angiographically documented coronary atherosclerotic lesions, whereas the control group had no angiographic evidence of coronary atherosclerosis. Coronary atherosclerotic burden was quantified using the Gensini score based on angiographic findings.

Inclusion criteria were as follows: (i) age 18–80 years; (ii) ability to provide written informed consent; and (iii) availability of EDTA‐anticoagulated peripheral blood for PBMC isolation and flow cytometry. For group allocation, participants were assigned to the AS group if coronary angiography showed definite atherosclerotic plaque with luminal stenosis in at least one major epicardial coronary artery or major branch; participants were assigned to the control group if coronary angiography showed no identifiable atherosclerotic plaque or stenotic lesions. All human procedures complied with the Declaration of Helsinki and were approved by the Institutional Review Board of The First Affiliated Hospital of Guangzhou University of Chinese Medicine (No. K‐2025‐165). Written informed consent was obtained.

### 4.14 Human PBMC Preparation and Flow Cytometry

Peripheral blood was collected in EDTA tubes. After red blood cell lysis, samples were centrifuged (400 × g, 5 min, 4°C), washed with cold Flow Cytometry Staining Buffer, and adjusted to ∼1 × 10^7^ cells/mL. Aliquots (100 µL; ∼1 × 10^6^ cells) were stained with CD14‐APC (clone M5E2, Elabscience; E‐AB‐F1209E) at 4°C for 20–30 min. Cells were then fixed/permeabilized and incubated with Anti‐c‐Rel/REL (Boster; A01880‐1), followed by Goat anti‐Rabbit IgG (H+L), FITC (Invitrogen; F‐2765), and analysed by flow cytometry.

### 4.15 Single‐Cell RNA Sequencing of Aortic Samples

Single‐cell libraries were constructed using the 10x Genomics Chromium platform and sequenced on an Illumina NovaSeq 6000 system. Raw sequencing data were processed with Cell Ranger (10x Genomics, v6.1.2) for quality control, alignment to the mm10 reference genome, and UMI counting to generate gene expression matrices for downstream analyses.

### 4.16 Single‐Cell RNA‐seq Processing and CD72^hi^ Macrophages Signal Analysis

scRNA‐seq of mouse aortas was completed previously and re‐analysed in this study using a unified R‐based workflow. For human validation, the carotid atherosclerosis scRNA‐seq dataset GSE159677 was downloaded from the Gene Expression Omnibus (GEO), which comprised three atherosclerotic plaque samples and three paired plaque‐adjacent arterial tissues. Expression matrices were imported into R using hdf5r and processed with Seurat. Two‐dimensional visualization was generated using UMAP.

Cell‐type annotation was conducted with SingleR using the ImmGen reference and refined based on canonical marker genes together with CD72 expression. Macrophages were identified and subsequently stratified into CD72^hi^ and CD72^low^ subsets based on CD72 expression within the macrophage compartment. CD72 expression levels were extracted on a per‐sample basis, and the relative abundance of CD72^hi^ macrophages was quantified.

Differential expression analysis was performed using the presto package, and results were visualized using violin plots and heatmaps. Cell–cell communication was inferred using a ligand–receptor–based framework, and predicted signaling interactions were compared between CD72^hi^ and CD72^low^ macrophages and endothelial cells, monocytes, smooth muscle cells, and fibroblasts. All analyses and visualizations were performed in R, with Seurat serving as the core analytical framework.

### 4.17 Transcriptome Analysis and Data Processing

Bulk RNA‐seq data derived from in vitro co‐culture systems and animal models were subjected to quality control with FastQC and trimming of low‐quality reads with Trimmomatic. Reads were aligned to the mouse reference genome (mm10) using HISAT2, and gene‐level expression matrices were generated with featureCounts. Differentially expressed genes (DEGs) were identified using DESeq2 (v1.30.0), with thresholds set at |log2FC| > 1 and adjusted *p* < 0.05. Functional annotation and pathway enrichment analyses of DEGs were performed using Gene Ontology (GO) and Kyoto Encyclopedia of Genes and Genomes (KEGG). GSEA was conducted with clusterProfiler (v4.0) to systematically evaluate the enrichment of biological processes related to inflammation, cell death, and immune regulation. Heatmaps of gene expression profiles were generated using the pheatmap package to visualize the expression patterns of key genes under different experimental conditions.

### 4.18 Transcriptome‐Based Compound Screening

A transcriptome‐based screening strategy previously established by our group was used to identify natural compounds with potential anti‐pyroptotic and anti‐atherosclerotic activities. Briefly, 102 plant‐derived compounds were analysed using normalized gene expression matrices from compound‐treated and DMSO control samples. For each compound, differential expression analysis was performed against the DMSO group, and genes were ranked according to the t‐statistic of expression changes.

Pyroptosis‐ and atherosclerosis‐related activation and suppression gene sets were curated from published literature. GSEA was then performed to evaluate whether each compound could reverse disease‐related transcriptional signatures. Pyroptosis Reversal Score (PRS) and Atherosclerosis Reversal Score (ARS) were calculated by integrating the normalized enrichment scores of the corresponding gene sets, and their standardized mean was defined as the Combo score. Compounds with high PRS and ARS values were identified by Pareto front analysis, among which IK was selected for further validation.

### 4.19 Molecular Docking and Molecular Dynamics Simulations

Molecular docking was performed using AutoDock Vina. The three‐dimensional structure of IK was retrieved from PubChem and energy‐minimized in Chem3D. The crystal structure of CXCR4 was downloaded from the Protein Data Bank (PDB) and processed to remove water molecules and ligands. Docking grids were defined at the active site of the protein, and docking was carried out to evaluate binding energies and conformations. Docking scores and predicted binding poses were used to assess the potential binding affinity and interaction modes of IK with CXCR4.

The top‐ranked complex from docking was subjected to molecular dynamics (MD) simulations. The complex was preprocessed in PyMol, and ligand topology files were generated with Sobtop under the AMBER force field. GROMACS (2020.3‐MODIFIED) was used to build the protein topology, with the amber99sb‐ildn.ff force field selected. The system was solvated with the TIP3P water model and neutralized with counter‐ions. Preparation included three stages: (1) energy minimization with the steepest descent algorithm for 5000 steps; (2) NVT equilibration with positional restraints while heating to 300 K for 100 ps with a 2 fs time step; and (3) NPT equilibration for 100 ps at 1 bar and 300 K. Subsequently, a 50 ns production MD simulation was performed without restraints.

Trajectory analysis was carried out using GROMACS utilities, including calculation of root mean square deviation (RMSD), root mean square fluctuation (RMSF), radius of gyration (Rg), hydrogen bond count, and free energy landscape, to evaluate the stability and dynamic properties of the IK‐CXCR4 complex.

### 4.20 Surface Plasmon Resonance Analysis

SPR analysis was performed using a Biacore system to determine the binding affinity of IK for CXCR4. Recombinant mouse CXCR4 protein was immobilized on a CM5 sensor chip by amine coupling. The chip surface was activated with EDC/NHS, followed by injection of CXCR4 protein diluted in sodium acetate buffer. After immobilization, residual active groups were blocked with ethanolamine. A blank reference channel was prepared using the same activation and blocking procedure without protein immobilization.

IK was dissolved in DMSO and further diluted in running buffer to the indicated concentrations. The final DMSO concentration was kept identical in all samples and running buffer. IK solutions were injected over the CXCR4‐immobilized surface at a constant flow rate, and the association and dissociation signals were recorded. The sensor surface was regenerated between injections when required. The resulting sensorgrams were processed by subtracting the reference‐channel and blank‐injection responses. Binding kinetics were fitted using a 1:1 Langmuir binding model, and the equilibrium dissociation constant was calculated using the Biacore evaluation software.

### 4.21 Pathological Assessment

Mice were anesthetized with sodium pentobarbital (50 mg/kg) and euthanized. Aortas, aortic roots, liver tissues, and kidney tissues were harvested, fixed, and processed for histological analysis. Atherosclerotic plaques were assessed in aortic root sections stained with Oil Red O. Plaque morphology, plaque area, and necrotic core size were evaluated by haematoxylin and eosin (HE) staining. Collagen content and fibrosis were examined using Masson's trichrome and Sirius Red staining, respectively. To evaluate potential tissue toxicity, liver and kidney sections were also subjected to H&E staining and examined for histopathological alterations. All images were acquired and analysed using ImageJ.

### 4.22 Lipid and Inflammatory Cytokine Measurement

Serum levels of TC, TG, low‐density lipoprotein (LDL), and high‐density lipoprotein (HDL) were measured using enzymatic colorimetric kits (Nanjing Jiancheng Bioengineering Institute, Nanjing, China; Cat# A110‐1‐1, A111‐1‐1, A113‐1‐1, A112‐1‐1). Inflammatory cytokines (IL‐1β, IL‐6, IL‐18, TNF‐α) were quantified using ELISA kits (Elabscience Biotechnology Co., Ltd., Wuhan, China). All assays were performed in duplicate according to manufacturer instructions.

### 4.23 Quantitative Real‐Time PCR

Total RNA was isolated from aortic tissues using TRIzol reagent (15596026CN, Invitrogen, USA) following the manufacturer's instructions. The purity and concentration of the extracted RNA were assessed by spectrophotometry. One microgram of total RNA from each sample was subsequently reverse‐transcribed into complementary DNA (cDNA) using the OneScript Plus cDNA Synthesis Kit (G236, Abm, Canada). Quantitative real‐time PCR was performed on a QuantStudio 5 Real‐Time PCR System (Thermo Fisher Scientific, USA) using the SYBR Green Pro Taq HS Pre‐Mixed qPCR Kit III (AG11739, AGbio, China). The thermal cycling conditions were as follows: initial denaturation at 95°C for 30 s, followed by 40 cycles of denaturation at 95°C for 5 s and annealing/extension at 60°C for 30 s. This study evaluated the mRNA expression levels of the following genes: *Il1b, Il6, Tnf, Nlrp3, Casp1, Gsdmd, Ldlr, Pcsk9, Hmgcr* and *Srebf2*. The relative mRNA expression levels were calculated using the comparative 2–^ΔΔCt^ method, with *Gapdh* serving as the internal reference gene for normalization. The nucleotide sequences of the specific primers used are provided in Table 1.

**TABLE 1:** Primer sequence.

Gene	Primer Sequence (5′‐3′)
*Nlrp3*	F: AGG CTG CTA TCT GGA GGA ACT
	R: TAG ACT CCT TGG CGT CCT GA
*Gsdmd*	F: GAT CAA GGA GGT AAG CGG CA
	R: CAC TCC GGT TCT GGT TCT GG
*Casp1*	F: GCC TGG TCT TGT GAC TTG GA
	R: GTC ACC CTA TCA GCA GTG GG
*Il1b*	F: AGG AGA ACC AAG CAA CGA CA
	R: CTC TGC TTG TGA GGT GCT GA
*Tnf*	F: GAC GTG GAA CTG GCA GAA GA
	R: GGC TAC AGG CTT GTC ACT CG
*Il6*	F: TGC CTT CTT GGG ACT GAT GC
	R: GCA AGT GCA TCA TCG TTG TTC
*Il18*	F: ACC AAG TTC TCT TCG TTG AC
	R: CTT CAC AGA GAG GGT CAC AG
*Ldlr*	F: CCA ATC GAC TCA CGG GTT CA
	R: ACA GTG TCG ACT TCT CTA GGC
*Pcsk9*	F: GAA GCA CCT CCT TCA CGG TC
	R: TGA GCT GGC TGT GAG TTG AC
*Hmgcr*	F: CAT CCG TGT ACG AGT GCC TG
	R: CCT TGG ATC CCA CGC GGA
*Srebf2*	F: TCT GGT TTG TCC GGT CTT CG
	R: CCA AAG TTT TCC AGA GCG CC
*Gapdh*	F: TGG ATT TGG ACG CAT TGG TC
	R: TTT GCA CTG GTA CGT GTT GAT

### 4.24 Statistical Analysis

Data are expressed as mean ± SD. Comparisons among multiple groups were performed using one‐way one‐way analysis of variance followed by Tukey's post hoc test (GraphPad Prism 9.0). Statistical significance was set at *p* < 0.05.

## Author Contributions

**Xingling He**: data curation, software, visualization, writing – original draft, writing – review and editing, formal analysis. **Sijing Li**: visualization, data curation, writing – original draft, formal analysis. **Jiahui Chen**: software, data curation, validation, writing – original draft. **Liyu Lin**: writing – original draft, validation, data curation, software. **Xinyu Li**: data curation, software, investigation. **Xiaojiao Zhang**: data curation, software, investigation. Yihui Zhang: software, data curation, investigation. **Xingling Chen**: software, data curation. Jinjian Guo: supervision, project administration. **Zhongqi Yang**: supervision, funding acquisition, writing – review and editing, project administration, resources. **Lu Lu**: software, supervision, funding acquisition, writing – review and editing, project administration, conceptualization, resources, methodology. **Shihao Ni**: conceptualization, methodology, supervision, funding acquisition, writing – review and editing, project administration, software, resources.

## Conflicts of Interest

The authors declare no conflicts of interest.

## Supporting information




**Supporting File**: advs77982‐sup‐0001‐SuppMat.docx.

## Data Availability

The data that support the findings of this study are available from the corresponding author upon reasonable request.

## References

[1] G. A. Roth , G. A. Mensah , C. O. Johnson , et al., “Global Burden of Cardiovascular Diseases and Risk Factors, 1990–2019: Update From the GBD 2019 Study,” Journal of the American College of Cardiology 76 (2020): 2982–3021, 10.1016/j.jacc.2020.11.010. PMC7755038 33309175

[2] S. Xu , Y. Liu , M. Zhu , K. Chen , F. Xu , and Y. Liu , “Global Burden of Atherosclerotic Cardiovascular Disease Attributed to Lifestyle and Metabolic Risks,” Science China Life Sciences 68, no. 9 (2025): 2739–2754, 10.1007/s11427-025-2948-y. 40553416

[3] GBD 2021 Causes of Death Collaborators , “Global Burden of 288 Causes of Death and Life Expectancy Decomposition in 204 Countries and territories and 811 Subnational Locations, 1990–2021: A Systematic Analysis for the Global Burden of Disease Study 2021,” Lancet 403 (2024), 2100–2132, 10.1016/S0140-6736(24)00367-2. PMC11126520 38582094

[4] F. Gomez‐Delgado , M. Raya‐Cruz , N. Katsiki , J. Delgado‐Lista , and P. Perez‐Martinez , “Residual Cardiovascular Risk: When Should We Treat It?,” European Journal of Internal Medicine 120 (2024): 17–24, 10.1016/j.ejim.2023.10.013. 37845117

[5] A. W. Aday and P. M. Ridker , “Targeting Residual Inflammatory Risk: A Shifting Paradigm for Atherosclerotic Disease,” Frontiers in Cardiovascular Medicine 6 (2019): 16, 10.3389/fcvm.2019.00016. PMC6403155 30873416

[6] M. A. Gimbrone and G. García‐Cardeña , “Endothelial Cell Dysfunction and the Pathobiology of Atherosclerosis,” Circulation Research 118, no. 4 (2016): 620–636, 10.1161/CIRCRESAHA.115.306301. PMC4762052 26892962

[7] V. Perrone , D. Sangiorgi , S. Buda , and L. Degli Esposti , “Residual Cardiovascular Risk in Patients Who Received Lipid‐lowering Treatment in a Real‐life Setting: Retrospective study,” ClinicoEconomics and Outcomes Research 8 (2016), 649–655, 10.2147/CEOR.S107992. PMC5087588 27822076

[8] N. D. Wong , Y. Zhao , R. G. W. Quek , et al., “Residual Atherosclerotic Cardiovascular Disease Risk in Statin‐Treated Adults: The Multi‐Ethnic Study of Atherosclerosis,” Journal of clinical lipidology 11, no. 5 (2017): 1223–1233, 10.1016/j.jacl.2017.06.015. PMC6394854 28754224

[9] P. Libby and O. Soehnlein , “Inflammation in Atherosclerosis: Lessons and Therapeutic Implications,” Immunity 58, no. 10 (2025): 2383–2401, 10.1016/j.immuni.2025.09.012. 41045921

[10] H. Xu , J. Jiang , W. Chen , W. Li , and Z. Chen , “Vascular Macrophages in Atherosclerosis,” Journal of Immunology Research 2019 (2019): 1–14, 10.1155/2019/4354786.

[11] I. Tabas and K. E. Bornfeldt , “Macrophage Phenotype and Function in Different Stages of Atherosclerosis,” Circulation Research 118, no. 4 (2016): 653–667, 10.1161/CIRCRESAHA.115.306256. PMC4762068 26892964

[12] L. Zhang , J. Li , Y. Kou , et al., “Mechanisms and Treatment of Atherosclerosis: Focus on Macrophages,” Frontiers in Immunology 15 (2024): 1490387, 10.3389/fimmu.2024.1490387. PMC11576186 39569201

[13] L. I. Susser and K. J. Rayner , “Through the Layers: How Macrophages Drive Atherosclerosis Across the Vessel Wall,” Journal of Clinical Investigation 132, no. 9 (2022): 157011, 10.1172/JCI157011. PMC9057606 35499077

[14] P. Hou , J. Fang , Z. Liu , et al., “Macrophage Polarization and Metabolism in Atherosclerosis,” Cell Death & Disease 14, no. 10 (2023): 691, 10.1038/s41419-023-06206-z. PMC10589261 37863894

[15] M. Piollet , F. Porsch , G. Rizzo , et al., “TREM2 Protects From Atherosclerosis by Limiting Necrotic Core Formation,” Nature Cardiovascular Research 3, no. 3 (2024): 269–282, 10.1038/s44161-024-00429-9. PMC7616136 38974464

[16] M. T. Patterson , Y. Xu , H. Hillman , et al., “Trem2 Agonist Reprograms Foamy Macrophages to Promote Atherosclerotic Plaque Stability—Brief Report,” Arteriosclerosis, Thrombosis, and Vascular Biology 44, no. 7 (2024): 1646–1657, 10.1161/ATVBAHA.124.320797. PMC11208052 38695172

[17] N.‐M. Popa‐Fotea , C.‐E. Ferdoschi , and M.‐M. Micheu , “Molecular and Cellular Mechanisms of Inflammation in Atherosclerosis,” Frontiers in Cardiovascular Medicine 10 (2023): 1200341, 10.3389/fcvm.2023.1200341. PMC10434786 37600028

[18] A. Lin , J. M. Miano , E. A. Fisher , and A. Misra , “Chronic Inflammation and Vascular Cell Plasticity in Atherosclerosis,” Nature Cardiovascular Research 3, no. 12 (2024): 1408–1423, 10.1038/s44161-024-00569-y. 39653823

[19] E. Mahdinia , N. Shokri , A. T. Taheri , S. Asgharzadeh , M. Elahimanesh , and M. Najafi , “Cellular Crosstalk in Atherosclerotic Plaque Microenvironment,” Cell Communication and Signaling 21, no. 1 (2023): 125, 10.1186/s12964-023-01153-w. PMC10227997 37254185

[20] R. Pandit and A. Yurdagul , “The Atherosclerotic Plaque Microenvironment as a Therapeutic Target,” Current Atherosclerosis Reports 27, no. 1 (2025): 47, 10.1007/s11883-025-01294-y. PMC11965263 40172727

[21] A. Ijaz , B. Yarlagadda , and M. Orecchioni , “Foamy Macrophages in Atherosclerosis: Unraveling the Balance Between Pro‐ and Anti‐Inflammatory Roles in Disease Progression,” Frontiers in Cardiovascular Medicine 12 (2025): 1589629, 10.3389/fcvm.2025.1589629. PMC12081418 40384967

[22] P. Theofilis , E. Oikonomou , K. Tsioufis , and D. Tousoulis , “The Role of Macrophages in Atherosclerosis: Pathophysiologic Mechanisms and Treatment Considerations,” International Journal of Molecular Sciences 24, no. 11 (2023): 9568, 10.3390/ijms24119568. PMC10253295 37298518

[23] C. Cochain , E. Vafadarnejad , P. Arampatzi , et al., “Single‐Cell RNA‐Seq Reveals the Transcriptional Landscape and Heterogeneity of Aortic Macrophages in Murine Atherosclerosis,” Circulation Research 122, no. 12 (2018): 1661–1674, 10.1161/CIRCRESAHA.117.312509. 29545365

[24] D. M. Fernandez , A. H. Rahman , N. F. Fernandez , et al., “Single‐cell Immune Landscape of human Atherosclerotic Plaques,” Nature Medicine 25, no. 10 (2019): 1576–1588, 10.1038/s41591-019-0590-4. PMC7318784 31591603

[25] S.‐H. Ni , J.‐D. Xu , S.‐N. Sun , et al., “Single‐cell Transcriptomic Analyses of Cardiac Immune Cells Reveal That Rel‐Driven CD72‐Positive Macrophages Induce Cardiomyocyte Injury,” Cardiovascular Research 118, no. 5 (2022): 1303–1320, 10.1093/cvr/cvab193. 34100920

[26] C. Zhang , Y. Zhang , Y. Yu , et al., “Macrophage‐Endothelial Cell Crosstalk Drives Atherosclerotic Plaque Formation and Progression,” European Journal of Pharmacology 1003 (2025): 177879, 10.1016/j.ejphar.2025.177879. 40582480

[27] L. Yu , L. Xu , H. Chu , et al., “Macrophage‐to‐Endothelial Cell Crosstalk by the Cholesterol Metabolite 27HC Promotes Atherosclerosis in Male Mice,” Nature Communications 14 (2023): 4101, 10.1038/s41467-023-39586-z. PMC10368733 37491347

[28] X. Liu , X. Liu , P. Luo , et al., “Roles of Pyroptosis in Atherosclerosis Pathogenesis,” Biomedicine & Pharmacotherapy = Biomedecine & Pharmacotherapie 166 (2023): 115369, 10.1016/j.biopha.2023.115369. 37643484

[29] B. Huang , Z. Zou , Y. Li , et al., “Gasdermin D‐Mediated Pyroptosis Promotes the Development of Atherosclerosis,” Laboratory Investigation 104, no. 4 (2024): 100337, 10.1016/j.labinv.2024.100337. 38266921

[30] Y. Lv , Z. Jiang , W. Zhou , et al., “Low‐Shear Stress Promotes Atherosclerosis via Inducing Endothelial Cell Pyroptosis Mediated by IKKε/STAT1/NLRP3 Pathway,” Inflammation 47, no. 3 (2024): 1053–1066, 10.1007/s10753-023-01960-w. PMC11147929 38315275

[31] R. C. Coll , K. Schroder , and P. Pelegrín , “NLRP3 and Pyroptosis Blockers for Treating Inflammatory Diseases,” Trends in Pharmacological Sciences 43, no. 8 (2022): 653–668, 10.1016/j.tips.2022.04.003. 35513901

[32] Y. Liu , R. Pan , Y. Ouyang , et al., “Pyroptosis in Health and Disease: Mechanisms, Regulation and Clinical perspective,” Signal Transduction and Targeted Therapy 9, no. 1 (2024): 245, 10.1038/s41392-024-01958-2. PMC11413206 39300122

[33] L. Zhong , X. Tan , W. Yang , et al., “Bioactive Matters Based on Natural Product for Cardiovascular Diseases,” Smart Materials in Medicine 5, no. 4 (2024): 542–565, 10.1016/j.smaim.2024.11.001.

[34] X. Chang , T. Zhang , W. Zhang , Z. Zhao , and J. Sun , “Natural Drugs as a Treatment Strategy for Cardiovascular Disease Through the Regulation of Oxidative Stress,” Oxidative Medicine and Cellular Longevity 2020 (2020): 1–20, 10.1155/2020/5430407. PMC7537704 33062142

[35] F. Feng , M. X. Zhu , N. Xie , W. Y. Liu , D. J. Chen , and Q. D. You , “Two New Triterpenoid Saponins From the Root of Ilex pubescens,” Journal of Asian Natural Products Research 10 (2008): 71–75, 10.1080/10286020701273874. 18058383

[36] S.‐H. Ni , X.‐J. Zhang , X.‐L. OuYang , et al., “Lobetyolin Alleviates Ferroptosis of Skeletal Muscle in 5/6 Nephrectomized Mice via Activation of Hedgehog‐GLI1 Signaling,” Phytomedicine 115 (2023): 154807, 10.1016/j.phymed.2023.154807. 37121057

[37] J.‐P. Deng , X. Liu , Y. Li , et al., “Drug Vector Representation and Potential Efficacy Prediction Based on Graph Representation Learning and Transcriptome Data: Acacetin From Traditional Chinese Medicine Model,” Journal of Ethnopharmacology 305 (2023): 115966, 10.1016/j.jep.2022.115966. 36572325

[38] S.‐H. Ni , X.‐L. OuYang , X. Liu , et al., “A Molecular Phenotypic Screen Reveals That Lobetyolin Alleviates Cardiac Dysfunction in 5/6 Nephrectomized Mice by Inhibiting Osteopontin,” Phytomedicine 107 (2022): 154412, 10.1016/j.phymed.2022.154412. 36191549

[39] L. Yu , Y. Zhang , C. Liu , et al., “Heterogeneity of Macrophages in Atherosclerosis Revealed by Single‐Cell RNA Sequencing,” The FASEB Journal 37, no. 3 (2023): 22810, 10.1096/fj.202201932RR. 36786718

[40] L. Willemsen and M. P. de Winther , “Macrophage Subsets in Atherosclerosis as Defined by Single‐Cell Technologies,” The Journal of Pathology 250, no. 5 (2020): 705–714, 10.1002/path.5392. PMC7217201 32003464

[41] M. T. Patterson , M. M. Firulyova , Y. Xu , et al., “Trem2 Promotes Foamy Macrophage Lipid Uptake and Survival in Atherosclerosis,” Nature Cardiovascular Research 2, no. 11 (2023): 1015–1031, 10.1038/s44161-023-00354-3. PMC11031198 38646596

[42] K. Miyake , J. Ito , K. Takahashi , et al., “Single‐cell Transcriptomics Identifies the Differentiation Trajectory From Inflammatory Monocytes to Pro‐resolving Macrophages in a Mouse Skin Allergy Model,” Nature Communications 15, no. 1 (2024): 1666, 10.1038/s41467-024-46148-4. PMC10891131 38396021

[43] J. R. Parnes and C. Pan , “CD72, a Negative Regulator of B‐Cell Responsiveness,” Immunological Reviews 176 (2000): 75–85, 10.1034/j.1600-065x.2000.00608.x. 11043769

[44] T. Adachi , H. Flaswinkel , H. Yakura , M. Reth , and T. Tsubata , “Cutting Edge: The B Cell Surface Protein CD72 Recruits the Tyrosine Phosphatase SHP‐1 Upon Tyrosine Phosphorylation,” The Journal of Immunology 160, no. 10 (1998): 4662–4665, 10.4049/jimmunol.160.10.4662. 9590210

[45] M. K. Galuppo , E. de Rezende , F. L. Forti , et al., “CD100/Sema4D Increases Macrophage Infection by Leishmania (Leishmania) amazonensis in a CD72 Dependent Manner,” Frontiers in Microbiology 9 (2018): 1177, 10.3389/fmicb.2018.01177. PMC5996280 29922261

[46] X. Song , J. Xue , E. Su , et al., “Endothelial Gasdermin D Induces Mitochondrial Damage and Activates the STING Pathway in Lipopolysaccharide‐Accelerated Atherosclerosis,” Antioxidants & Redox Signaling 44 (2026): 61–84, 10.1177/15230864251380286. 41017337

[47] X. Qiao , M. Ji , Y. Yao , et al., “Pubescenosides E–K, Seven New Triterpenoid Saponins From the Roots of Ilex Pubescens and Their Anti‐Inflammatory Activity,” Molecules 23, no. 6 (2018): 1426, 10.3390/molecules23061426. PMC6100132 29895796

[48] J. Chen , D. Cao , S. Jiang , et al., “Triterpenoid Saponins From Ilex Pubescens Promote Blood Circulation in Blood Stasis Syndrome by Regulating Sphingolipid Metabolism and the PI3K/AKT/eNOS Signaling Pathway,” Phytomedicine 104 (2022): 154242, 10.1016/j.phymed.2022.154242. 35728385

