# Supplementary material for: Ilexoside K Ameliorates Atherosclerosis by Suppressing CD72hi Macrophages and Blocking CXCL12–CXCR4 Axis to Inhibit Endothelial Pyroptosis
Source: Adv Sci (Weinh). 2026 Sep 27:e77982. Online ahead of print. doi: 10.1002/advs.77982 (PMC13616409; doi:10.1002/advs.77982)
Supplement: Supplementary file 1 [file ADVS-9999-e77982-s001.docx]

**Figure S1. CD72 expression and endothelial CASP1-associated activation in atherosclerosis and Rel-deficient mice.**

(A,B) Representative western blots of CD72 in aortic tissues from control and atherosclerotic (AS) mice (A), with densitometric quantification normalized to β-tubulin (B) (*n* = 3 per group).

(C,D) Quantification of relative CASP1 fluorescence intensity within CD31^+^ endothelial areas (C) and representative immunofluorescence images of aortic root sections stained for CD31 (red), CASP1 (green), and DAPI (blue) (D) from ApoE^-/-^ and Rel^-/-^ApoE^-/-^ mice (*n* = 5 per group). Scale bars, 10 μm.

(E,F) Representative western blots of CD72 in aortic tissues from ApoE^-/-^ and Rel^-/-^ApoE^-/-^ mice (E), with densitometric quantification normalized to β-tubulin (F) (*n* = 3 per group).

Data are presented as the mean ± SD. Each data point represents one mouse. Statistical analyses were performed as described in the Methods. ^**^*P* < 0.01; ^***^*P* < 0.001; ^****^*P* < 0.0001.

**Figure S2. Hematopoietic Rel deficiency reduces CD72-associated macrophage accumulation and attenuates atherosclerosis, systemic inflammation, and endothelial pyroptosis-associated activation.**

(A) Schematic illustration of the bone marrow transplantation (BMT) protocol. Lethally irradiated ApoE^-/-^ recipient mice received bone marrow cells from ApoE^-/-^ donors (Control_BMT) or Rel^-/-^ApoE^-/-^ donors (Rel_KO_BMT).

(B,C) Representative western blots of CD72 in aortic tissues from Control_BMT and Rel_KO_BMT mice (B), with densitometric quantification normalized to β-tubulin (C) (*n* = 3 per group).

(D,E) Quantification of CD72^+^F4/80^+^ macrophages in aortic cell suspensions (D) and representative flow cytometry plots showing CD72 and F4/80 expression (E) in Control_BMT and Rel_KO_BMT mice (*n* = 5 per group).

(F,G) Representative immunofluorescence images of aortic root sections stained for F4/80 (green), CD72 (red), and DAPI (blue) (F), together with quantification of CD72^+^F4/80^+^ macrophages in aortic root lesions (G) (*n* = 5 per group). Scale bars, 50 μm.

(H–J) Representative H&E, Masson’s trichrome, and Picro Sirius Red (PSR) staining of aortic root sections (H), with quantification of plaque area (I) and plaque collagen content (J) in the two BMT groups (*n* = 5 per group). Scale bars, 500 μm.

(K,L) Representative en face Oil Red O staining of whole aortas (K) and quantification of Oil Red O-positive aortic lesion area (L) (n = 4 per group).

(M–P) Serum levels of total cholesterol (M), triglycerides (N), LDL cholesterol (O), and HDL cholesterol (P) in Control_BMT and Rel_KO_BMT mice (*n* = 5 per group).

(Q–T) Serum concentrations of IL-1β (Q), TNF-α (R), IL-6 (S), and IL-18 (T) in the two BMT groups (*n* = 5per group).

(U,W) Quantification of relative CASP1 fluorescence intensity within CD31^+^ endothelial areas (U) and representative immunofluorescence images of aortic root sections stained for CD31 (red), CASP1 (green), and DAPI (blue) (W) (*n* = 5 per group). Scale bars, 10 μm.

(V,X) Quantification of relative GSDMD-N fluorescence intensity within CD31^+^ endothelial areas (V) and representative immunofluorescence images of aortic root sections stained for CD31 (red), GSDMD-N (green), and DAPI (blue) (X) (*n* = 5 per group). Scale bars, 10 μm.

**Figure S3. IK dose selection and complementary validation of CD72 expression and endothelial CASP1-associated activation.**

(A,B) Representative immunofluorescence images of aortic root sections stained for CD31 (red), CASP1 (green), and DAPI (blue) in control and atherosclerotic model mice (A), together with quantification of relative CASP1 fluorescence intensity within CD31^+^ endothelial areas (B) (*n* = 6 per group). Scale bars, 50 μm.

(C) CCK-8-based viability of RAW264.7 macrophages treated with increasing concentrations of Ilexoside K (IK; 0–100 μM) (*n* = 6 per group).

(D) CCK-8-based viability of RAW264.7 macrophages exposed to ox-LDL in the absence or presence of increasing concentrations of IK (*n* = 6 per group).

(E,F) Representative western blots of CD72 in aortic tissues from the control, atherosclerosis (AS), low-dose IK (IK-L), high-dose IK (IK-H), and atorvastatin (ATO) groups (E), with densitometric quantification normalized to β-tubulin (F) (*n* = 3 group).

(G) Representative immunofluorescence images of aortic root sections stained for CD31 (red), CASP1 (green), and DAPI (blue), together with quantification of relative endothelial CASP1 fluorescence intensity in the five experimental groups (*n* = 8 per group). Scale bars, 20 μm.

**Figure S4. Comparison of regulated cell death programs and endothelial p-MLKL signaling in Rel-deficient models.**

(A) Single-sample gene set enrichment analysis (ssGSEA) scores for nine regulated cell death-related programs—apoptosis, necroptosis, pyroptosis, ferroptosis, autophagy-related cell death, cuproptosis, disulfidptosis, parthanatos, and PANoptosis—in MAECs co-cultured with CD72^low^ macrophages, CD72^hi^ macrophages, or CD72^hi^ macrophages plus IK (*n* = 3 per group). Colored points represent individual biological replicates, and black diamonds and connecting lines indicate group means.

(B,C) Representative immunofluorescence images of aortic root sections stained for CD31 (red), p-MLKL (green), and DAPI (blue) in Control_BMT and Rel_KO_BMT mice (B), together with quantification of relative endothelial p-MLKL fluorescence intensity (C) (*n* = 5 per group). Scale bars, 10 μm

(D,E) Representative immunofluorescence images of aortic root sections stained for CD31 (red), p-MLKL (green), and DAPI (blue) in ApoE^-/-^ and Rel^-/-^ApoE^-/-^ mice (D), together with quantification of relative endothelial p-MLKL fluorescence intensity (E) (*n* = 5 per group). Scale bars, 10 μm.

**Figure S5. Adoptive transfer of Rel-overexpressing monocytes partially restores endothelial CASP1-associated activation during IK treatment.**

(A) Representative immunofluorescence images of aortic root sections stained for CD31 (red), CASP1 (green), and DAPI (blue), together with quantification of relative CASP1 fluorescence intensity within CD31^+^ endothelial areas in the control, atherosclerotic model, IK-treated, and Rel-overexpressing monocyte transfer (Rel_Mo) groups (*n* = 8 per group). Rel_Mo denotes IK-treated mice receiving adoptive transfer of Rel-overexpressing monocytes. Scale bars, 10 μm.

**Figure S6. CD72-associated macrophages exhibit increased CXCL12 expression in atherosclerotic lesions.**

(A–C) Representative immunofluorescence images of aortic root sections stained for F4/80 (red), CD72 (magenta), CXCL12 (green), and DAPI (blue) in ApoE^-/-^ and Rel^-/-^ApoE^-/-^ mice (A), together with quantification of CD72^+^F4/80^+^ macrophages (B) and relative CXCL12 fluorescence intensity (C) in aortic root lesions (*n* = 5 per group). Scale bars are indicated in the images.

(D) Relative *Cxcl12* mRNA expression in isolated CD72^low^ and CD72^hi^ macrophages (*n* = 6 per group).

(E) CXCL12 concentrations in culture supernatants from isolated CD72^low^ and CD72^hi^ macrophages (*n* = 6 per group).

(F–H) Representative immunofluorescence images of aortic root sections stained for F4/80 (red), CD72 (magenta), CXCL12 (green), and DAPI (blue) in the control, atherosclerosis (AS), IK-treated, and Rel-overexpressing monocyte transfer (Rel_Mo) groups (F), together with quantification of CD72^+^F4/80^+^ macrophages (G) and relative CXCL12 fluorescence intensity (H) in aortic root lesions (*n* = 6 per group). Rel_Mo denotes IK-treated mice receiving adoptive transfer of Rel-overexpressing monocytes. Scale bars, 20 μm.

**Figure S7. Endothelial CXCR4 overexpression attenuates IK-associated reductions in endothelial inflammasome activation without substantially altering CD72 expression or hepatic lipid-metabolism responses.**

(A,B) Representative western blots of CD72 in aortic tissues from the AAV-NC, AAV-CXCR4, AAV-NC + IK, and AAV-CXCR4 + IK groups (A), with densitometric quantification normalized to β-tubulin (B) (*n* = 3 per group).

(C,E) Representative immunofluorescence images of aortic root sections stained for CD31 (red), CASP1 (green), and DAPI (blue) (C), together with quantification of relative CASP1 fluorescence intensity within CD31^+^ endothelial areas (E) in the four experimental groups (*n* = 8 per group). Scale bars, 10 μm.

(D,F) Representative immunofluorescence images of aortic root sections stained for CD31 (red), NLRP3 (green), and DAPI (blue) (D), together with quantification of relative NLRP3 fluorescence intensity within CD31^+^ endothelial areas (F) in the four experimental groups (*n* = 8 per group). Scale bars, 10 μm.

(G,H) Hepatic total cholesterol (G) and triglyceride ( H) contents normalized to total protein in the four experimental groups (*n* = 8 per group).

(I–L) Relative hepatic mRNA expression of *Pcsk9* (I), *Ldlr* (J), *Hmgcr* (K), and *Srebf2* (L) in the four experimental groups (*n* = 8 per group).

AAV-NC, mice receiving the control AAV vector; AAV-CXCR4, mice receiving the endothelial CXCR4-overexpression vector; AAV-NC + IK, mice receiving the control AAV vector and IK treatment; AAV-CXCR4 + IK, mice receiving the endothelial CXCR4-overexpression vector and IK treatment.
